# Dissecting Structure-Encoded Determinants of Allosteric Cross-Talk between Post-Translational Modification Sites in the Hsp90 Chaperones

**DOI:** 10.1038/s41598-018-25329-4

**Published:** 2018-05-02

**Authors:** Gabrielle Stetz, Amanda Tse, Gennady M. Verkhivker

**Affiliations:** 1 Department of Computational and Data Sciences, Schmid College of Science and Technology, Chapman University, Orange, California United States of America; 2 Chapman University School of Pharmacy, Irvine, California United States of America

## Abstract

Post-translational modifications (PTMs) represent an important regulatory instrument that modulates structure, dynamics and function of proteins. The large number of PTM sites in the Hsp90 proteins that are scattered throughout different domains indicated that synchronization of multiple PTMs through a combinatorial code can be invoked as an important mechanism to orchestrate diverse chaperone functions and recognize multiple client proteins. In this study, we have combined structural and coevolutionary analysis with molecular simulations and perturbation response scanning analysis of the Hsp90 structures to characterize functional role of PTM sites in allosteric regulation. The results reveal a small group of conserved PTMs that act as global mediators of collective dynamics and allosteric communications in the Hsp90 structures, while the majority of flexible PTM sites serve as sensors and carriers of the allosteric structural changes. This study provides a comprehensive structural, dynamic and network analysis of PTM sites across Hsp90 proteins, identifying specific role of regulatory PTM hotspots in the allosteric mechanism of the Hsp90 cycle. We argue that plasticity of a combinatorial PTM code in the Hsp90 may be enacted through allosteric coupling between effector and sensor PTM residues, which would allow for timely response to structural requirements of multiple modified enzymes.

## Introduction

Significant biological insights in the functional roles of diverse PTM types in regulation of protein families and signaling networks have been obtained in recent years by utilizing systems-level quantitative analyses, including high-resolution mass spectrometry (MS) and systems biology approaches^1–6^. According to these large-scale investigations, PTMs can regulate protein activity through diverse mechanisms, including modifications of binding sites and protein-protein interactions, protein localization, degradation, cleavage, and allosteric regulation of enzyme activity. Systematic functional analysis of 200,000 phosphorylation, acetylation, and ubiquitination sites from 11 eukaryotic species prioritized the functional relevance of PTMs in cross-regulatory events and protein-protein interactions by considering evolutionary conservation patterns of PTMs within domain families^5^. Diversity and sensitivity of PTM-mediated regulation is enabled not only by individual PTM sites and specific PTM types, but often through cooperative action and mutual interdependencies between multiple PTMs, which is often referred as functional PTM cross-talk^6–8^. Multiple PTMs can be linked through evolutionary and functional couplings that can be used to distinguish PTM-mediated regulatory hotspots and molecular switches of protein functions and allosteric interactions^6^. A comparative analysis of multiple PTM types in different eukaryotic species revealed correlated evolution of PTM pairs in proteins, showing that coevolving PTM pairs are not necessarily close in sequence space but can often enjoy structural proximity and physical interactions, forming regulatory centers in protein structures^7^. A proteome-wide survey of co-occurring phosphorylation pairs that tend to be modified together showed that these PTMs can be often functionally associated^8^. Using large experimental data sets, another study identified motifs enriched by pairs of known PTM sites, including three combinations of PTM types: phosphorylation-acetylation, phosphorylation-SUMOylation, and phosphorylation-phosphorylation^9^. A systematic characterization of functional cross-talk between PTM sites using 193 experimentally validated PTM pairs in 77 human proteins demonstrated that these pairs could exhibit proximity in both sequence and structure space, showing preferential colocalization for flexible and disordered regions^10^. Integration of acetylation, ubiquitination and phosphorylation datasets with protein interaction data emphasized a central role of phosphorylation in mediating of protein–protein interactions^11^. Among 12 different main PTM types and across 9 diverse species, phosphorylated proteins feature the broadest spectrum of physically interacting proteins in signaling networks^12^. MS-based proteomics studies revealed complexity of functional cross-talk between PTMs in protein families, suggesting that a combinatorial PTM code may be at play to enable additional layers of protein communication, biological regulation and redundancies in the cellular environment^13–16^.

Structure-centric studies of PTMs in protein families showed that residues modified by more than one type of PTM can often correspond to more disordered regions than single PTM sites^17^. The majority of PTMs are typically localized in protein structural environments accessible to outside modifying enzymes, mostly in solvent-exposed and flexible regions^18^ and the accessibility criterion can be used to triage the proposed PTM sites^19^. Phosphorylation events are often accompanied by conformational switching of proteins that triggers significant changes in the accessibility of PTM sites, suggesting that these modifications of these functional residues can enact allosteric transformations in protein structures^20–22^. Computational analysis of phospho-proteome quantified structural preferences of PTM sites, showing that a significant amount of studied phosphorylation sites (24.6%) can reside in relatively inaccessible protein regions^23^. Phosphorylation and lysine acetylation sites tend to be enriched at the protein interfacial regions and have a significant impact on protein function by modulating the strength and specificity of physical binding interactions in protein networks^24,25^. A systematic analysis of PTM sites that can alter protein functions by promoting allosteric conformational changes suggested that allosteric PTMs can play an important role in plasticity of a combinatorial PTM code, allowing for precise regulatory control of multiple PTMs in the proteome context^26^.

Dissecting functional roles and couplings of PTMs in the family of the 90 kDa heat-shock (Hsp90) proteins is of fundamental and therapeutic importance as these abundant and evolutionary conserved molecular chaperones manage conformational development, maturation and folding for a wide array of protein client substrates, including protein kinases and transcription factors^27–30^. The Hsp90 chaperone operates as a homodimer and each monomer consists of three distinct domains: an N-terminal domain (Hsp90-NTD) that harbors the ATP binding site, a middle domain (Hsp90-MD), which is implicated in binding of client proteins, and a C-terminal domain (Hsp90-CTD) that is required for constitutive dimerization. Structural studies characterized main stages of the Hsp90-ATPase functional cycle associated with the ATP binding and hydrolysis, in which Hsp90 progresses from open apo and ADP-bound forms to a catalytically competent closed ATP-bound dimer^28–30^. The Hsp90 functions and interactions with client substrates can be regulated by cochaperones^31,32^ and through a diverse spectrum of PTMs, including phosphorylation, acetylation, *S*-nitrosylation, methylation, oxidation, ubiquitination, and SUMOylation^33,34^. Several phosphorylation sites in the Hsp90-NTD (T22 andY24 in yeast Hsp82) can alter progression of the ATPase cycle and modulate activation of protein kinase clients by weakening interactions with the kinase-specific cochaperone Cdc37 and impeding conformational transitions between functional states^35–37^. A quantitative analysis of phosphorylation sites in yeast Hsp82 revealed their functional role as molecular switches of conformational equilibrium and allosteric interactions as phosphorylation-mimicking chaperone variants in the Hsp82-MD regions (S379E, S485E) and Hsp82-CTD (S602E, S604E) featured the markedly reduced rates of ATP hydrolysis^38^. A greater number of phosphorylation sites were identified in the human Hsp90α and Hsp90β chaperones^39,40^. A systematic survey of PTM sites in molecular chaperones argued that these sites may have been selected by evolution for rapid and efficient regulation of diverse chaperone functions due to their integrating role in cellular protein networks^41^. Although decoding PTM patterns and linking them with specific regulatory mechanisms presents a daunting task, there are several lines of evidence suggesting that cochaperones and PTMs can exploit similar mechanisms to modulate chaperone function^42^. Recent evidence suggested that inhibition of client kinases that catalyze the vast majority of Hsp90 PTMs can act synergistically with Hsp90 inhibitors, providing a novel therapeutic strategy to enhance the efficacy of Hsp90 inhibitors in cancer cells^43,44^.

The large number of PTM sites in the Hsp90 proteins that are scattered throughout all domains (sew Supplementary Tables S1–S3) indicated that synchronization of multiple PTMs through a combinatory code can be invoked as a mechanism to orchestrate diverse chaperone functions and recognize multiple substrates with high fidelity^35–40^. Despite recent progress in functional characterization of PTMs in protein systems, molecular mechanisms by which functionally important PTMs can exert control over allosteric regulation in the Hsp90 chaperones remain poorly understood and mostly qualitative in nature. In the current study, we have combined evolutionary analysis, all-atom and coarse-grained simulations of the Hsp90 proteins with the network analysis of residue interactions and perturbation response scanning (PRS) approach to probe allosteric mechanisms and quantify functional roles of PTM sites in allosteric regulation of the molecular chaperone. This study provides a comprehensive structural, dynamic and network analysis of PTM sites across Hsp90 proteins, identifying specific role of regulatory PTM hotspots in modulating of allosteric interactions and signal transmission in the Hsp90 structures. The results reveal conserved PTMs that act as central mediators and regulatory hotspots of allosteric signaling in the Hsp90 proteins, while the majority of PTM sites serve as sensors and transmitters of the allosteric signal in collectively moving Hasp90 regions. By combining PRS analysis of functional centers and network-based modeling of allosteric communication pathways, we determine key regulatory PTM sites in the Hsp90 chaperones can serve as gate-keepers and conformational switches of chaperone functions and interactions.

## Results and Discussion

### Evolutionary Conservation and Spatial Distribution of PTM Sites in the Hsp90 Proteins are linked to Functional Roles

**Figure 1:**
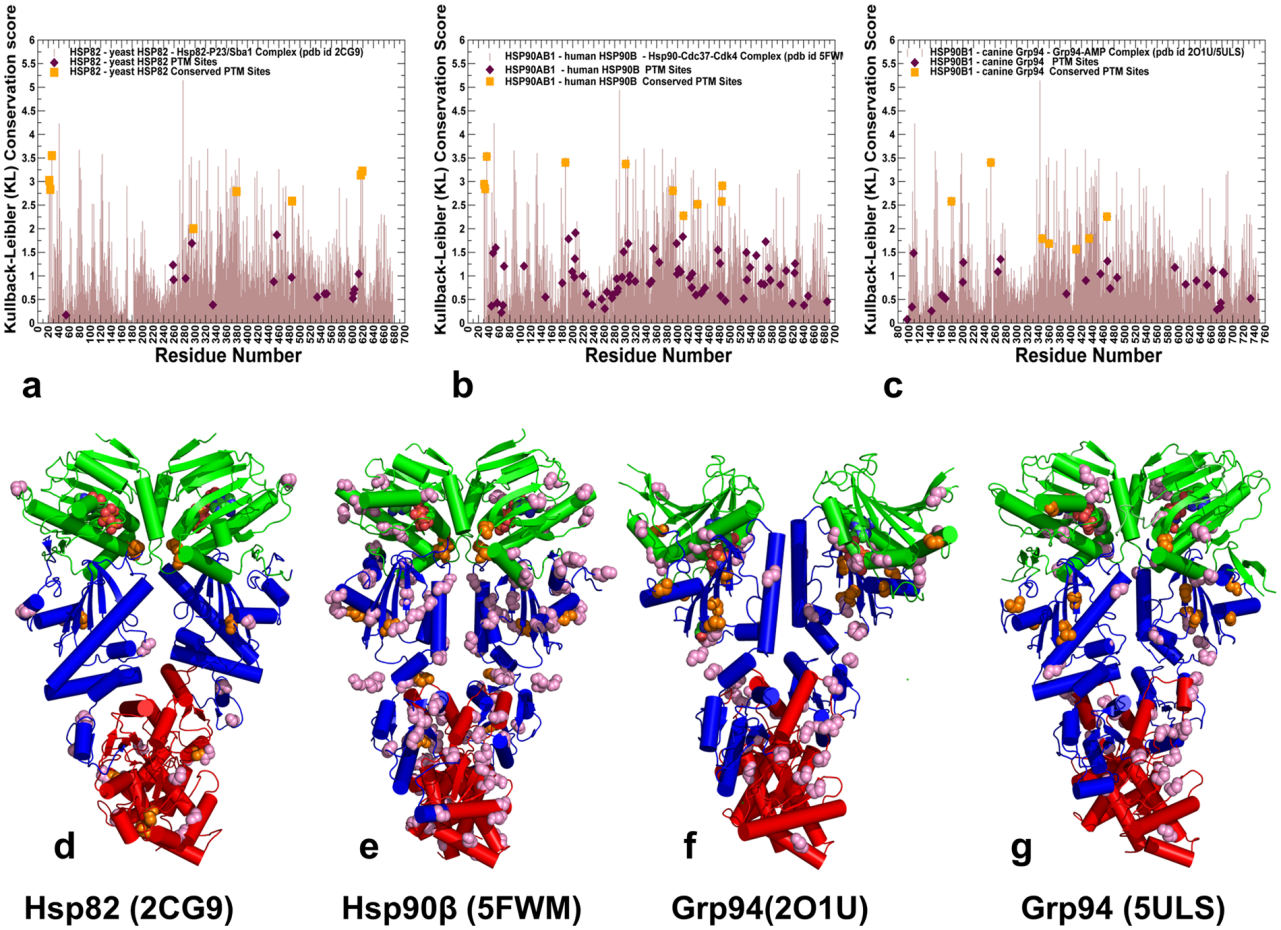
Evolutionary Analysis and Structural Organization of the Hsp90 Proteins. The KL conservation score of the Hsp90 proteins was calculated using MSA profiles of the HATPase_c family (PF02518), corresponding to the Hsp90-NTD residues and the Hsp90 family (PF00183) that corresponds to the Hsp90-MD and CTD domains. The KL score is mapped onto the Hsp90 residues in the structures of ATP-bound yeast Hsp82 (**a**), human Hsp90β in the Hsp90-Cdc37-Cdk4 complex (**b**), and ATP-bound forms of canine Grp94 (**c**). The KL conservation profiles are shown in brown bars, the position of experimentally known PTM sites are annotated as filled maroon diamonds, and the conserved PTM sites are shown as filled orange squares. (**d**) The crystal structure of ATP-bound yeast Hsp82 (pdb id 2CG9) is shown in a ribbon representation and the domains are colored as follows: Hsp90-NTD (residues 2–216, in green), Hsp90-MD (residues 217–526, in blue) and Hsp90-CTD (residues 527–677, in red). (**e**) The cryo-EM structure of the human Hsp90β in the Hsp90-Cdc37-Cdk4 complex (pdb id 5FWM/5FWL). The domains are annotated as follows: NTD (residues 1–215, in green), MD (residues 216–552, in blue), and CTD (residues 553–690, in red). (**f**) The crystal structure of the canine Grp94 in the complex with AMPPNP (pdb id 2O1U). The domains are annotated as follows: NTD (residues 85–337, in green), MD (residues 338–593, in blue), and CTD (residues 594–749, in red). (**g**) The crystal structure of AMPPNP-bound Grp94 in the fully closed dimer conformation that additionally includes a region preceding the N-terminal domain, pre-NTD domain that is highly conserved in Grp94, but not in other Hsp90s (residues 48–72). In the panels (d–f), the residues corresponding to the experimentally known PTM sites are shown as pink spheres, and the evolutionary conserved PTM sites are highlighted as orange spheres.

To systematically characterize PTM sites in the Hsp90 chaperone proteins, we surveyed PhosphoSitePlus database (http://www.phosphosite.org/) that contains over 330,000 non-redundant PTMs, with 95% of the sites obtained from mass spectrometry experiments^3^. We extracted all PTM sites in the Hsp90 genes that emerged from the experimental studies with at least two validated references. The experimentally validated Hsp90 PTM sites that are examined in this work included yeast Hsp82 (see Supplementary Fig. S1 and Supplementary Table S1), human Hsp90β (see Supplementary Fig. S2 and Supplementary Table S2), and Grp94 chaperones (see Supplementary Fig. S3 and Supplementary Table S3). We began with the evolutionary analysis to identify conserved PTM sites in the Hsp90 proteins and characterize the sequence-based determinants of functionally significant PTM positions. The relative Shannon entropy (also called the Kullback–Leibler (KL) divergence score metric^45^ was used to evaluate sequence conservation in the Hsp90 chaperones (Fig. 1a–c). The KL conservation score was calculated in the framework of MISTIC approach^46^ using multiple sequence alignment (MSA) profile generated using hidden Markov models in the Pfam database^47^. To analyze domain-based distribution of PTM sites in the Hsp90 proteins, we mapped KL conservation scores with the respective protein residues in the crystal structure of ATP-bound yeast Hsp82 (pdb id 2CG9)^48^ (Fig. 1a), the cryo-electron microscopy (EM) structure of ATP-bound human Hsp90β (pdb id 5FWM)^49^ (Fig. 1b), and crystal structures of ATP-bound forms of canine Grp94 (pdb id 2O1U, 5ULS)^50,51^ (Fig. 1c). In the reported KL conservation score profiles, the higher KL values correspond to more conserved sites. By mapping KL values onto the residues in the Hsp90 crystal structures, we identified conserved PTM sites as the ones that displayed sequence conservation scores markedly higher than the average score (Fig. 1a–c). In agreement with the experimental studies of PTM sites, the vast majority of PTM sites in the Hsp90 proteins exhibited low-to-intermediate KL scores, consistent with their characteristic moderate conservation. Structural mapping of the PTM sites showed a broad distribution of PTM positions across Hsp90 domains (Fig. 1d–g), and these sites were primarily found in solvent-accessible regions, with the largest and most dense cluster of PTMs situated in the Hsp90-CTD regions. These results are consistent with previous studies that identified 145 phosphorylation sites in the Hsp90 chaperone and noted that PTMs were preferentially enriched in the Hsp90-CTD regions^5^. Of particular importance was the observed enrichment of PTM sites in CTD residues 600–620 (residue numbering in yeast Hsp82), as this region in all Hsp90 structures forms contacts with the equivalent segment of the opposing dimer. While the majority of PTM sites were localized in flexible regions accessible to modifying enzymes, a number of conserved PTM sites were partially buried, often occupying strategic positions near the inter-domain and dimerization interfaces (Fig. 1d–g).

In yeast Hsp82 protein, the conserved phosphorylation sites (T22, Y24, S297, S379, S485, S616 and S619) are spatially separated and occupy all three domains (Fig. 1a). Most of these PTM sites are functionally significant and have a strong effect on chaperone regulation and client binding^35–39^. In particular, mutations of the Hsp82-NTD sites (T22 andY24) have a profound effect on the ATPase cycle by impeding conformational transitions and preventing formation of a catalytically competent closed dimer^35–37^. According to recent functional studies, phosphorylation of S379 position can perturb local structural environment of the catalytic site and weaken the inter-domain interactions between NTD and MD regions^38^. At the same time, mutations of other conserved PTM sites (S485, S602, S604, S616, and S619) are believed to exert their deleterious effects by interfering with the allosteric cross-talk between PTMs and compromising long-range allosteric communications in the Hsp82 dimer^38^. A significantly larger number of known PTM sites in the human Hsp90β provided more convincing statistics, showing that the vast majority of PTM residues belonged to highly variable positions (Fig. 1b). Structural mapping of PTM sites in the human Hsp90β showed that these residues belonged mainly to solvent-exposed regions, with the high concentration of PTMs found in the Hsp90-MD and Hsp90-CTD regions (Fig. 1e).

The conserved PTMs in the human Hsp90β (T31, Y33, K36, K186, K286, Y301, S391, K411, K438, Y484, Y485) included several important phosphorylation sites (T31, Y33) and a number of lysine acetylation sites that populated the inter-domain regions (Fig. 1e). The highly conserved PTMs (T31, Y33, K186, Y301, S391) included functionally important sites that are targeted and phosphorylated by specific kinases, including Ck2 kinase (T31), Week1 kinase (Y33)^35^, c-Src kinase (Y301)^52^, and pregnancy*-*upregulated nonubiquitous calmodulin kinase Pnck (S391 site)^53,54^. Interestingly, phosphorylation status of Y301 in the Hsp90β chaperone can elicit isoform specific binding to therapeutic agent geldamacyn (GA), as phosphomimetic mutations in this position considerably reduce inhibitor binding, while the respective mutations in Hsp90α (Y309E and Y309F) did not have a measurable effect on the binding affinity^43^. Several moderately conserved PTM sites (K286, S365) in the human Hsp90β (Fig. 1b) have a significant regulatory role, indicating that evolutionary conservation of PTMs may not be a unique determinant of their functional significance^39,40^. Recent experiments revealed that isoform-specific phosphorylation at position S365 in the human Hsp90β affects client maturation and impairs progression of the Hsp90-kinase cycle by altering conformational equilibrium and disfavoring the closed dimer form^39^. Mutational analysis of K274 in the yeast Hsp82 (equivalent to K294 in Hsp90α and K286 in the Hsp90β) showed that changes in the acetylation state of PTM sites that are proximal to inter-domain interfaces can radically affect cochaperone binding and compromise maturation of client proteins^40^. While this site showed only a moderate sequence conservation, it is shared by yeast Hsp82 (K274) and Hsp90α (K294), indicating that functional importance and conservation of PTMs are not linked through a simple linear relationship. The conserved PTM positions in the Grp94 chaperone are exclusively lysine acetylation sites (K179, K252, S347, K360, K410, K434, and K467) that occupied the NTD and MD regions (Fig. 1c,f,g). Several conserved PTM sites in the Grp94-MD (K410, K434, and K467) occupy proximal structural positions near the NTD-MD interface regions and their communication may be relevant in allosteric regulation of the Grp94 chaperone^55^.

Hence, evolutionary and structural analyses suggested that functional PTM sites in the Hsp90 proteins tend to be conserved and often occupy strategic positions near the inter-domain interfaces (Fig. 1). However, specialized regulatory roles of PTM sites in modulating conformational changes and progression of the ATPase cycle cannot be readily inferred from sequence conservation patterns and require understanding of structural and dynamics mechanisms. Accordingly, in addition to evolutionary conservation patterns, we examined coevolutionary, structural and dynamic environments of PTM sites that shape up their specific regulatory roles and functional cross-talk in the Hsp90 proteins.

### Structural Environment of PTM Sites in the Hsp90 Proteins

**Figure 2:**
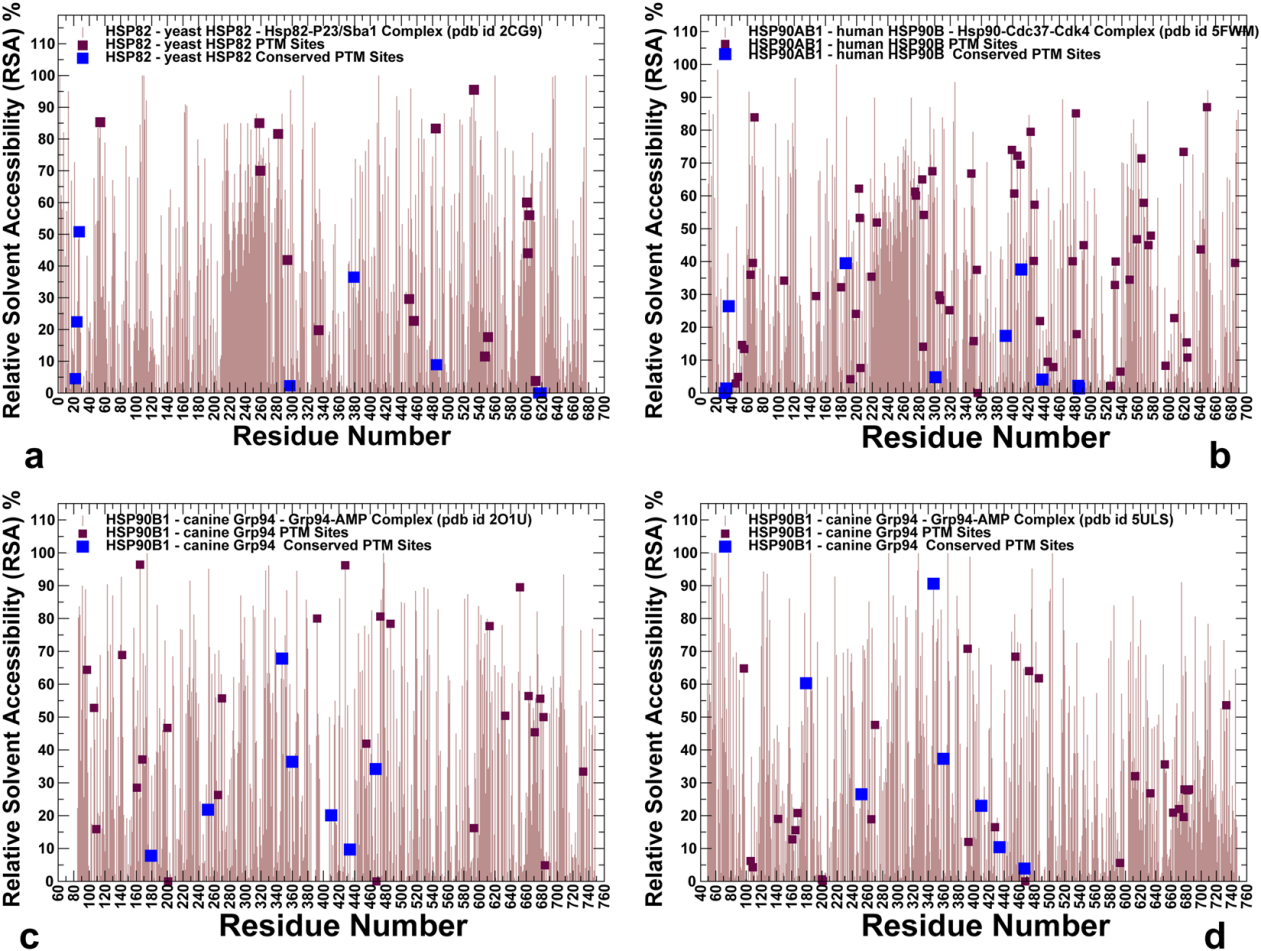
The Relative Solvent Accessibility Profiles of the Hsp90 Structures. The residue-based relative solvent accessibility RSA profiles (in light brown lines) are obtained by averaging computations over the equilibrium trajectories for structures of the yeast Hsp82 (**a**), human Hsp90β (**b**), and two different structures of canine Grp94 (**c**,**d**). A residue-specific local RSA measure is defined as the ratio of the observed solvent-accessible surface area for a residue to the expected unfolded state value for that amino acid type. The RSA values for residues corresponding to the experimentally known PTM sites are shown as filled maroon squares and the evolutionary conserved PTM sites are highlighted in filled magenta squares.

**Figure 3:**
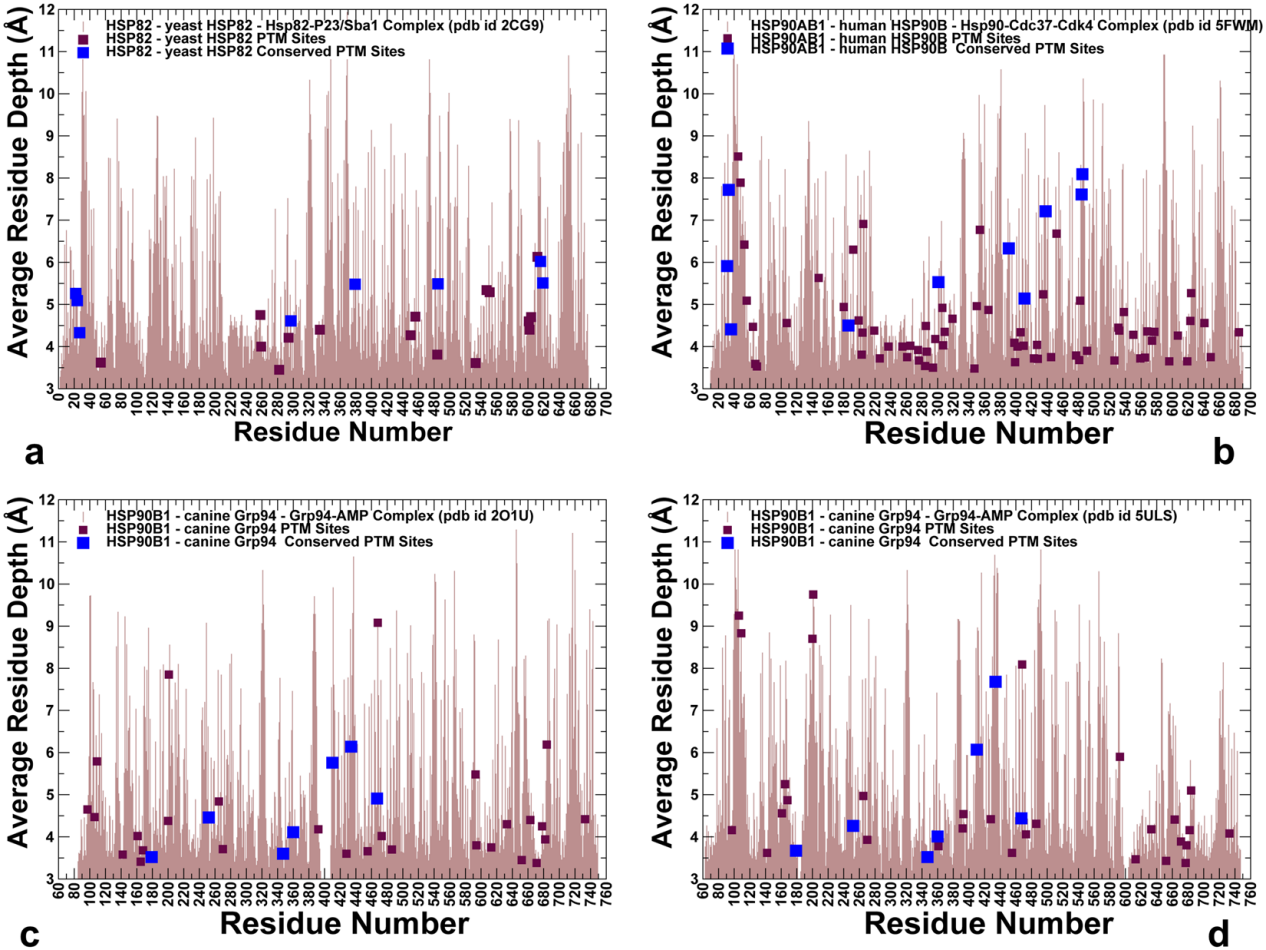
The Residue Depth Profiles of the Hsp90 Structures. The residue-based residue depth RD profiles (in light brown lines) are obtained by averaging computations over the equilibrium trajectories for structures of the yeast Hsp82 (**a**), human Hsp90β (**b**), and two different structures of canine Grp94 (**c**,**d**). RD parameter measures the closest distance of the residue to bulk solvent. The RD values for residues corresponding to the experimentally known PTM sites are shown as filled maroon squares and the evolutionary conserved PTM sites are highlighted in filled magenta squares.

Solvent accessibility and conformational flexibility are often strong indicators of PTM sites in proteins. However, conformational transformations in allosterically regulated systems can change structural environment and modulate the exposure and accessibility of the regulatory PTM sites. To characterize structural environment of PTM sites in the Hsp90 proteins, we evaluated relative solvent accessibility (RSA) of protein residues (Fig. 2) and residue depth (RD) (Fig. 3) in the Hsp90 structures. A residue-specific local RSA measure is defined as the ratio of the observed solvent-accessible surface area for a residue to the expected unfolded state value for that amino acid type^56,57^. The RSA values can be used as proxy for predicting intrinsic residue flexibility and the extent of conformational changes that may occur upon complex formation. According to this model, residues are considered to be completely solvent exposed if the ratio value exceeds 50% and to be buried if the ratio is less than 20%.

By mapping all PTM sites on the RSA profiles and highlighting conserved PTM positions, we observed a significant variability in solvent exposure (Fig. 2). In general, evolutionary conserved PTM sites displayed only moderate accessibility and were often partially buried at the Hsp90 dimer interface (within 10–40% RSA). At the same time, several moderately conserved PTM sites could reside in a partially buried environment. While most of the phosphorylation sites in yeast Hsp82 tend to have high RSA values and are associated with solvent accessible regions, some of the functionally important phosphorylation sites (T22, Y24, S485) showed only a moderate degree of RSA < 25% (Fig. 2a). Interestingly, most of the conserved regulatory phosphorylation sites become partially buried in the closed form of yeast Hsp82 (Fig. 2a). Conserved PTM sites in the Hsp90β (T31, Y33, K36, Y301, S391, K411, K438, Y484, Y485) also displayed relatively small RSA values and are located in poorly accessible regions of the closed chaperone dimer (Fig. 2b). Notably, some of these PTM sites (T31, Y33, Y301, and S391) can be phosphorylated by specific kinases^35,52–54^. These regions are involved in coordinated structural changes that render the respective PTM sites become accessible to modifications by kinase clients. While structural changes can radically alter accessibility status for some PTM sites in the open and closed Grp94 structures, only moderate RSA differences were detected for conserved K410, K434, and K467 positions that retained their relatively low accessibility in both structural forms of Grp94 (Fig. 2c,d). The observed variations in solvent accessibility of the conserved PTM sites may serve as indicators of their involvement in functional motions and potential regulatory role in the allosteric mechanism.

Another relevant parameter to evaluate variations in solvent exposure of the PTM sites is the residue depth (RD), which determines not only whether a residue is exposed or buried, but also quantifies the distance (depth) of a residue from the protein surface and from the closest bulk water^58,59^. This parameter correlates with protein stability changes, reproducing residue packing density and hydrogen-exchange profiles^60^. For solvent-exposed regions in the Hsp90 proteins, RSA and RD parameters followed the same trend and predicted similar solvent accessible PTM sites (Figs 2 and 3). However, for buried residues, RD values were considerably more sensitive to the packing density in the respective region, better differentiating between partially and completely buried sites (see Supplementary Fig. S4). According to our analysis, only residues with very low RSA (RSA < 10%) would have large RD values and likely feature considerable structural stability (see Supplementary Fig. S4). As expected, the bulk of PTM sites displayed relatively small RD values (RD < 3.0) in all studied Hsp90 structures (Fig. 3). Of particular interest were moderate RD values for conserved PTM sites in yeast Hsp82 (T22, Y24, S379, S485, S616, S619) (Fig. 3a). A generally similar trend was seen in Hsp90β, featuring somewhat larger RD values for solvent-insulated, conserved PTM sites in the Hsp90-MD regions (Fig. 3b). A comparative analysis of RD distributions in two different Grp94 structures showed the increased RD values (and reduced accessibility) for some PTM sites in the NTD/MD regions in the closed dimer form (Fig. 3c,d). Our analysis indicated that conserved PTM sites may be confined to a moderately accessible environment even in the tightly packed closed chaperone states. We argue that PTM sites can preferentially populate regions that undergo large conformational changes making these sites accessible either in a transient higher energy state or in an allosterically different state of similar energy.

### Conformational Dynamics of the Hsp90 Structures Links Differential Mobility of PTM Sites with Regulatory Functions

**Figure 4:**
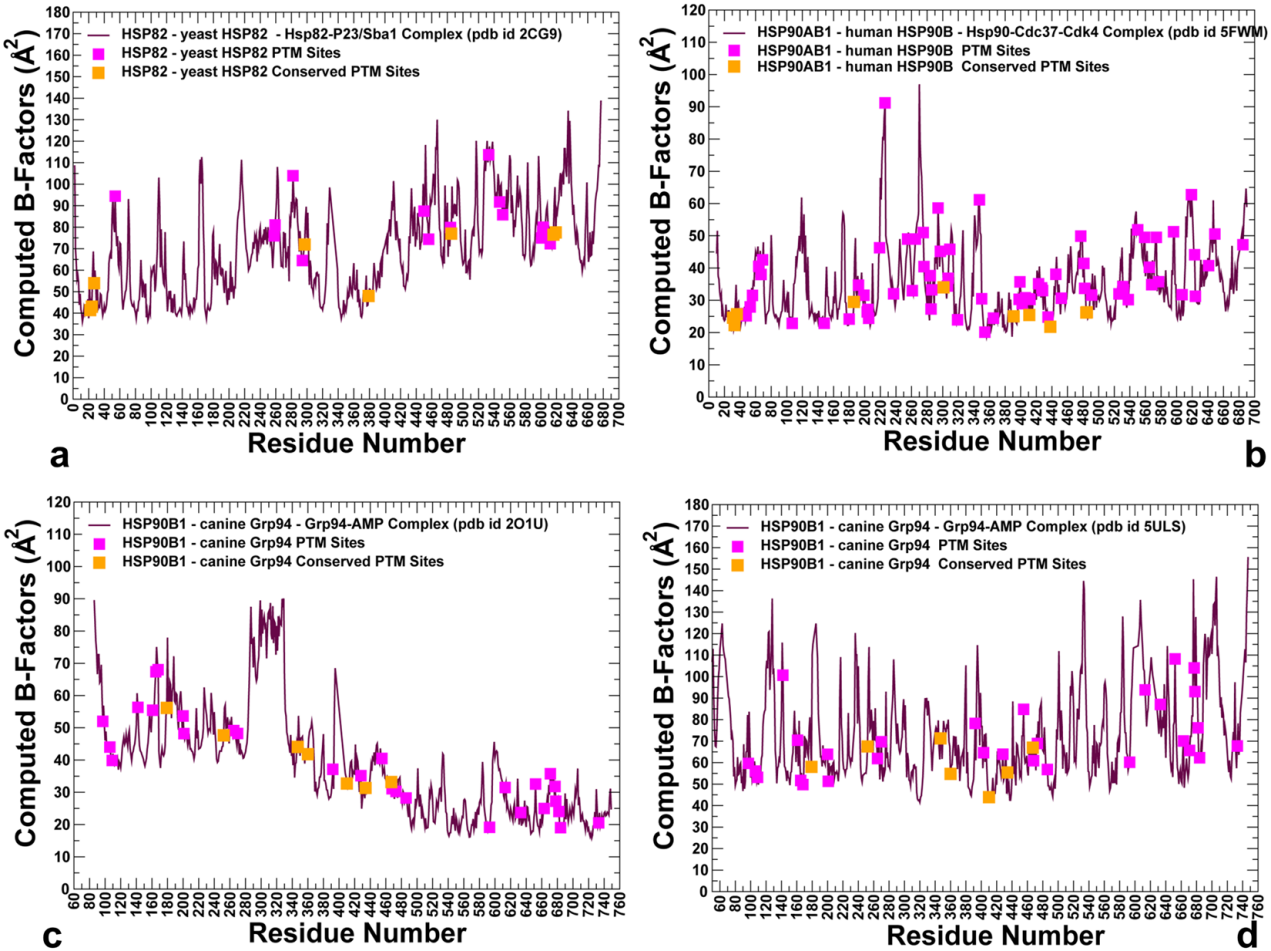
Conformational Dynamics Profiles of the Hsp90 Proteins from Atomistic MD Simulations. Conformational dynamics profiles obtained from all-atom MD simulations of the yeast Hsp82 (**a**), human Hsp90β (**b**), and canine Grp94 (**c**,**d**). Residue-based conformational mobility profiles are represented by computed B-factors for protein residues. The mobility values of PTM sites along profiles are depicted in filled magenta squares and evolutionary conserved PTM sites are highlighted in filled orange squares.

**Figure 5:**
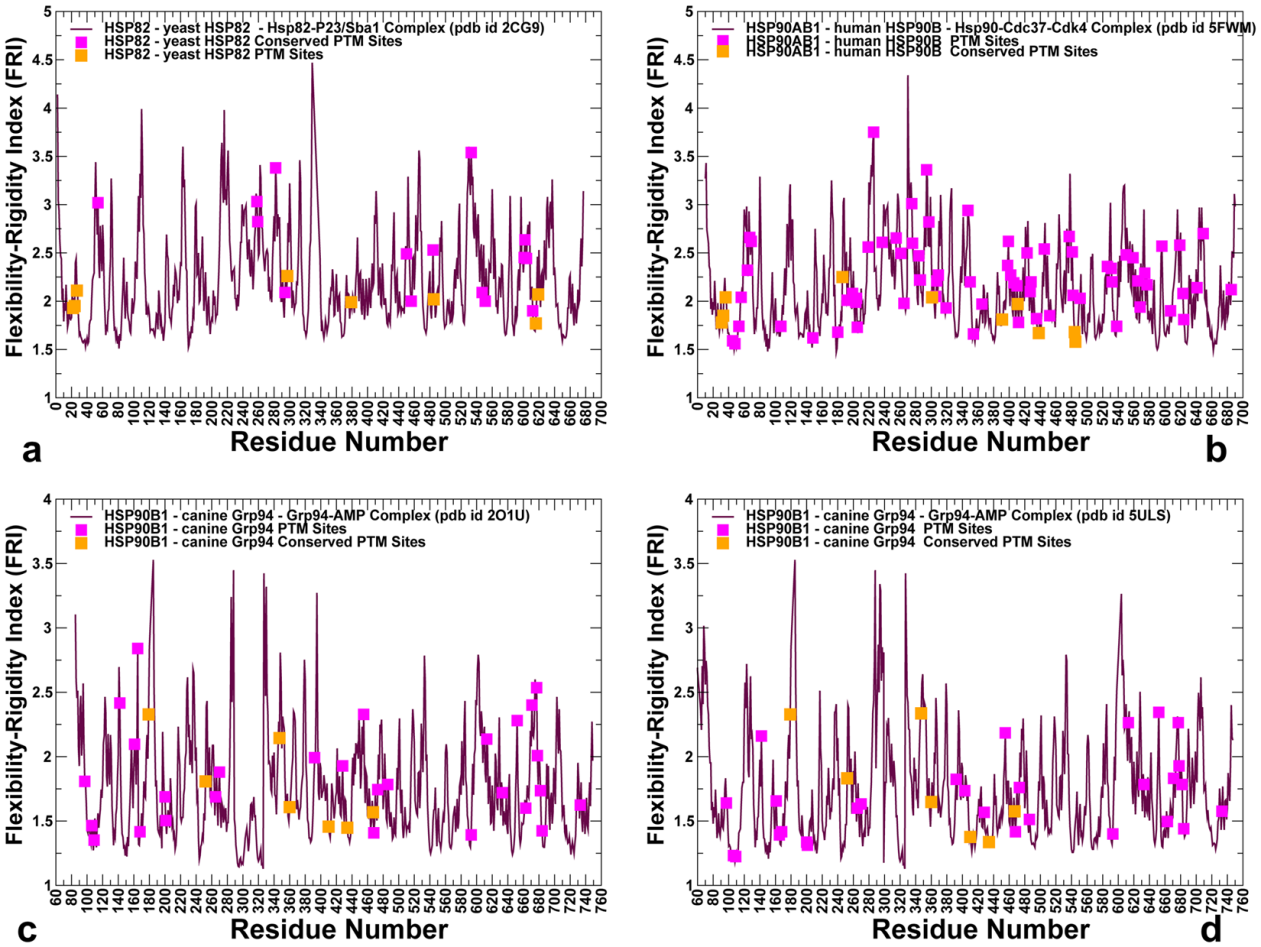
Conformational Mobility Analysis of the Hsp90 Proteins: Flexibility-rigidity FRI Profiles. The ensemble-based average FRI profiles are generated from the equilibrium DMD trajectories for structures of the yeast Hsp82 (**a**), human Hsp90β (**b**), and two different structures of canine Grp94 (**c**,**d**). The FRI values for PTM sites are shown filled magenta squares along the profiles and evolutionary conserved PTM sites are annotated in filled orange squares.

We conducted both atomistic molecular dynamics (MD) simulations and coarse-grained discrete molecular dynamics (DMD)^61–63^ of the Hsp90 structures. In these simulations, we explored and characterized conformational landscapes of ATP-bound yeast Hsp82 (pdb id 2CG9)^48^ (Fig. 1a), ATP-bound human Hsp90β (pdb id 5FWM)^49^ (Fig. 1b), and ATP-bound forms of canine Grp94 (pdb id 2O1U, 5ULS)^50,51^ (Fig. 1c). We focused on a comparative analysis of conformational variations in the Hsp90 structures obtained from all-atom MD simulations and coarse-grained DMD modeling. A series of independent 500 ns MD simulations was performed to characterize conformational dynamics and equilibrium ensembles of the Hsp90 structures. Conformational mobility of protein residues was evaluated by using the mean square residue fluctuations and computed B-factors (Fig. 4). In DMD simulations, the coarse-grained conformational ensembles of the Hsp90 structures were subjected to all-atom reconstruction^64,65^ and subsequent refinement^66^. We then applied flexibility-rigidity index (FRI) method^67,68^ which is a matrix decomposition-free method that utilizes topological connectivity in protein structures to estimate rigidity and flexibility of Hsp90 residues. Using all-atom reconstructed conformations along DMD trajectories, we computed the ensemble-based average FRI profiles for the Hsp90 structures (Fig. 5). Strikingly, conformational dynamics profiles of the Hsp90 structures derived using two different approaches and metric showed generally similar trends, particularly highlighting mobility patterns of the PTM sites (Figs 4 and 5). Conformational mobility of the ATP-bound forms of yeast Hsp82 (Fig. 4a) and human Hsp90β (Fig. 4b) were consistent with the HX-MS studies, showing the increased stability of the ATP site and the ATP-lid (residues 91–105/114–124 in yeast Hsp82 and residues 99–114/126–133 in human Hsp90β)^69,70^. The FRI profiles revealed a similar dynamic pattern, pointing to stabilization of the ATP binding site region (Fig. 5a,b). The dynamics profiles also reproduced conformational mobility of the client-binding Src-loop in the MD region (residues 329-FDLFESKKKKN-340 in the yeast Hsp82 and residues 341-FDLFENKKKK-350 in the Hsp90β chaperone)^71^. Conformational dynamics profiles (Fig. 4) and FRI indexes (Fig. 5) showed similar flexibility patterns, consistently highlighting the reduced mobility of conserved PTM sites as opposed to the higher average flexibility of the majority of PTM positions. In the closed dimer of yeast Hsp82, several prominent PTM sites important for chaperone regulation (T22, Y24, S379) displayed structural rigidity according to atomistic simulations (Fig. 4a) and coarse-grained modeling (Fig. 5a). A similar picture emerged for the human Hsp90β dimer, where conserved PTM residues (T31, Y33, Y301, S391, K411) that can be phosphorylated by specific kinases displayed reduced mobility and are partially protected from solvent in the closed dimer conformation (Figs 4b and 5b). The respective positions in Hsp90α (T36, Y38, S399, and K419) also occupied low mobility regions and emerged as important communication hubs of allosteric interactions^72^. One of these PTM sites (S379 in Hsp82, S391 in Hsp90β and S399 in Hsp90α respectively) belongs to a conserved SRE motif of the catalytic loop, which is involved in the nucleotide coordination and stabilization of the NTD-MD interactions. These results suggested that strategic structural position and stability of the conserved PTM sites in the NTD and NTD-MD regions may be associated with their unique regulatory roles. At the same time, we noticed that PTM sites in the CTD regions may form local clusters of highly mobile residues. In particular, a number of Hsp90β PTM sites (K550, K559, K564, K568, K574 and K577) corresponded to local fluctuation peaks in the dynamics profile (Fig. 4b) and displayed larger FRI indexes (Fig. 5b). Interestingly, the corresponding PTM sites in human Hsp90α (K558, K567, K573, K576, K582, and K585) similarly displayed large thermal fluctuations in MD simulations of Hsp90α homology models^72^, confirming the elevated mobility level and local clustering of PTM sites in the CTD regions.

Conformational dynamics profiles of the partially open and closed Grp94 dimers reflected structural differences between these forms (Fig. 4c,d). In the open Grp94 structure, the NTD regions experience large movements, while the Grp94-CTD is mostly rigid (Fig. 4c). Indeed, even a partial truncation of the peripheral CTD regions may lead to a considerable loss of the hydrolysis activity^50,51,55^. In the open Grp94 form, conserved PTM positions in the NTD regions (K179, K252, S347, K360) maintained considerable mobility, while another group of regulatory PTM sites (K410, K434, and K467) displayed a markedly reduced mobility and only moderate accessibility (Fig. 4c). In the closed Grp94 dimer, all conserved PTMs displayed only moderate mobility, whereas a significant fraction of solvent-exposed PTM sites in the CTD featured markedly larger fluctuations (Fig. 4d). A generally similar picture emerged from the FRI analysis (Fig. 5c,d), particularly highlighting low mobility of the PTM sites in the Grp94-MD regions (K410, K434, and K467). Collectively, atomistic and coarse-grained simulations revealed a consistent view of conformational dynamics in the Hsp90 structures, highlighting structural and dynamic separation of PTM positions in the Hsp90 proteins. According to our findings, a small group of conserved and stable PTM sites near the NTD-MD regions can be contrasted with a larger group of accessible and mobile PTM residues near the CTD regions.

To quantify the relationship between structural and dynamic features of the Hsp90 proteins, we also constructed scatter plots between RSA, RD and FRI profiles (see Supplementary Fig. S5). In general, the correlation between residue depth and flexibility index follows the exponential function (see Supplementary Fig. S5). Only when RD > 5.0 residues showed a gradual increase in stability, indicating that only a small fraction of PTM sites would correspond to stable regions while the majority of PTM positions would enjoy some flexibility and display an appreciable solvent exposure. We observed that RD difference is not significant when solvent accessibility is high, but when RSA is low and residue becomes buried, RD values can be different and better quantify the extent of residue burial and stability. The variations in RD values may arise from differences in residue orientation and accessibility of side-chain conformations. According to our analysis, only residues with very low RSA (RSA < 10%) would have larger RD values and display a considerable stability.

### Functional Dynamics of the Hsp90 Structures: Conserved Regulatory PTM Sites Mediate Cooperative Functional Motions

**Figure 6:**
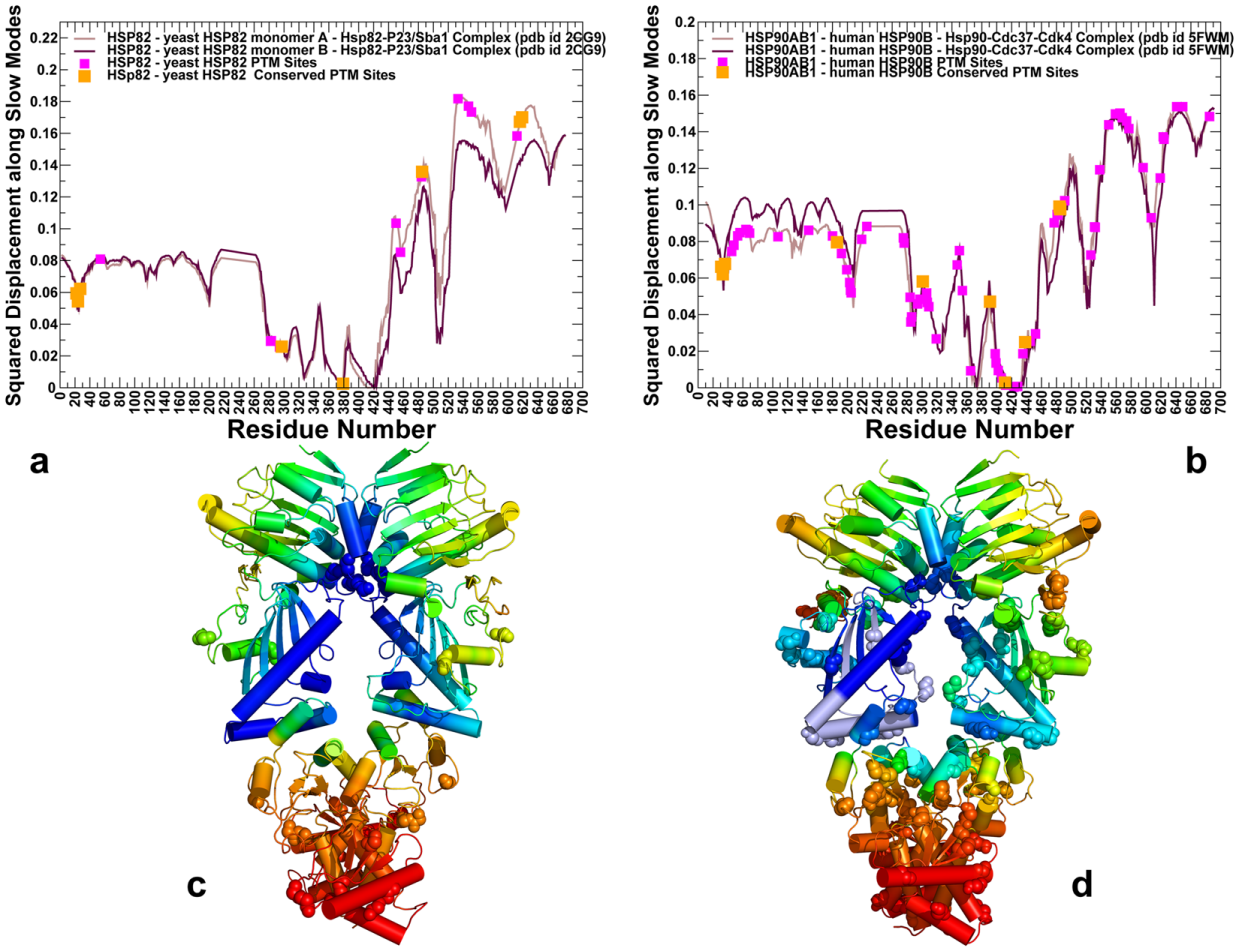
Functional Dynamics and Slow Mode Profiles of the yeast Hsp82 and human Hsp90β Dimers. Functional dynamics profiles of the yeast Hsp82 (**a**) and human Hsp90β (**b**) are represented as normalized square displacements of the protein residues averaged over the first three PCA modes. The residue-based slow mode profiles are shown for the monomer A (in light brown bars) and for the monomer B (in maroon bars). The PTM sites are mapped onto the slow modes profiles and depicted as filled magenta squares. The conserved PTM sites are annotated as filled orange squares. Structural mapping of the functional dynamics profiles driven by the slowest three PCA modes onto the crystal structure of ATP-bound Hsp82 dimer (**c**) and cryo-EM structure of the human Hsp90β dimer from the complex with Cdc37-Cdk4 (**d**). The color gradient from blue to red indicates the decreasing structural stability (or increasing conformational mobility) of protein residues. The PTM sites are shown as spheres colored according to their mobility in the slow modes.

To verify our hypothesis that regulatory PTM sites in the low mobility regions can mediate functional motions and collective dynamics of the Hsp90 structures, we utilized Principal Component Analysis (PCA)^73^ of DMD trajectories and analyzed residue displacements along the first three lowest frequency modes (Fig. 6). The maxima along the slow mode profiles correspond to regions undergoing global structural movements, while the local minima describe immobilized hinge positions that coordinate collective dynamic changes. The known regulatory PTM sites in the Hsp82-NTD (T22 and Y24) and S379 site from the catalytic loop corresponded to local minima, while other conserved phosphorylation sites (S485, S616 and S619) from the MD and CTD regions aligned closely with the distribution maxima (Fig. 6a). Hence, regulatory PTM sites (T22, Y24, and S379) can form global hinge centers that control collective movements in the Hsp90 dimer. As a result, phosphorylation of S379 can interfere not only with the local structural environment of the catalytic site and NTD-MD interactions, but also affect global conformational changes in the Hsp90 dimer^38^. Structural map of the functional dynamics profile showed a high concentration of PTM sites in the CTD regions (Fig. 6b), which may be associated with functional role of these residues as carriers of allosteric conformational changes. We argue that deleterious effects of phosphomimetic variants for several conserved PTM residues S485, S616, and S619 in the/CTD regions^38^ may result from the impaired allosteric coupling between the NTD and CTD regions, which is required for propagation of conformational changes during chaperone cycle.

A similar trend emerged from the analysis of the human Hsp90β (Fig. 6c,d). Strikingly, only few PTM positions (K286, S365 and K411) mapped onto local minima along the slow mode profiles, indicating that these residues can serve as hinge sites that coordinate functional movements of the Hsp90 dimer. Although S365 and K286 positions are not strongly conserved, the unique structural positions of these residues near the inter-domain and dimer interfaces contribute to their unique regulatory role in allosteric signaling. At the same time, a number of conserved PTM sites in the NTD and MD regions (K186, Y301, S391, K438, Y484, and Y485) corresponded to local maxima along the slow mode profile. Another major distribution peak corresponded to a local cluster of PTM sites (K550, K559, K564, K568, K574 and K577) in the CTD region that is involved in cooperative conformational changes during chaperone cycle. Interestingly, computational analysis of allosteric regulation in human Hsp90α pointed to similar elevated global mobility of respective residues (K558, K567, K573, K576, K582 and K585), confirming the role of this CTD region in modulating collective motions across Hsp90 proteins^72^.

In general, we found that moderately conserved PTM sites (a large fraction of PTMs) can serve as integrating nodes of collectively moving regions in global modes, while several highly conserved PTM positions (a small fraction of PTMs) belong to stable hinge centers that coordinate execution of allosteric transitions in the Hsp90 structures. Based on these results, we suggested that functional cross-talk between PTM sites may be associated with their role in modulating collective motions and allosteric communications in the Hsp90 structures.

We also explored the relationships between conformational dynamics, sequence conservation and residue coevolution in the Hsp90 protein family.

Using mutual information (MI) approach^46^, we computed cumulative mutual information score (cMI) for each residue and the proximity mutual information score (pMI), which measures the mutual information shared by the residues in the structural proximity of a given residue. The important observation of coevolutionary analysis is that conserved PTM sites near regulatory hinge centers of the NTD-MD region are characterized by high pMI values, while moderately conserved and flexible PTM residues in the CTD regions can be distinguished by high cMI values (see Supplementary Fig. S6). According to our results, the PTM sites residing in the cooperatively moving regions in global modes typically feature high cMI scores (see Supplementary Fig. S6).

As a result, these PTM sites can mediate coevolutionary network that confers a direct path for transmitting long-range signals between mobile regions. In this mechanism, local clusters of coevolving residues in the mobile CTD regions are enriched by PTM sites to control propagation of conformational changes and modulation of allosteric signaling in the Hsp90 dimers (See Supplementary Information online for extended discussion of coevolutionary analysis).

### Perturbation Response Scanning Specifies Functional Crosstalk between PTM Sites as Effectors and Sensors of Allosteric Signaling

We employed perturbation response scanning (PRS) method to quantify the role of PTM sites in allosteric signaling by probing the effect of each residue in the Hsp90 structures on all other residues in response to external perturbation. PRS formalism is rooted in the linear response theory to estimate residue response to external forces applied systematically to each residue in the protein system^74,75^. This approach was successfully used in probing allosteric mechanisms in single protein domains and large multi-domain assemblies^76–79^. The average perturbation response maps are matrices in which the *ij*th element refers to the average response (displacement) of residue *j* to external perturbation at residue *i*. The average effect of the perturbed effector site *i* on all other residues ${\langle {({\rm{\Delta }}{{\boldsymbol{R}}}^{{\boldsymbol{i}}})}^{2}\rangle }_{effector}$ is computed by averaging over all sensor residues $j$. The effector profile measures the ability of residue *i* to influence dynamics changes in all other residues. In this model, the maxima of the effector profiles can be interpreted as allosteric hotspot residues that exert global control over propagation of allosteric communications to other protein residues. The sensor profile ${\langle {(\Delta {{\boldsymbol{R}}}_{{\boldsymbol{j}}})}^{2}\rangle }_{sensor}$ measures the response of residue *j* to perturbations of all other residues and evaluates its ability to serve as a receiver (carrier) of allosteric signal. As a result, the maxima along the sensor profile can be attributed to residues that are involved in transmission of allosteric structural changes.

**Figure 7:**
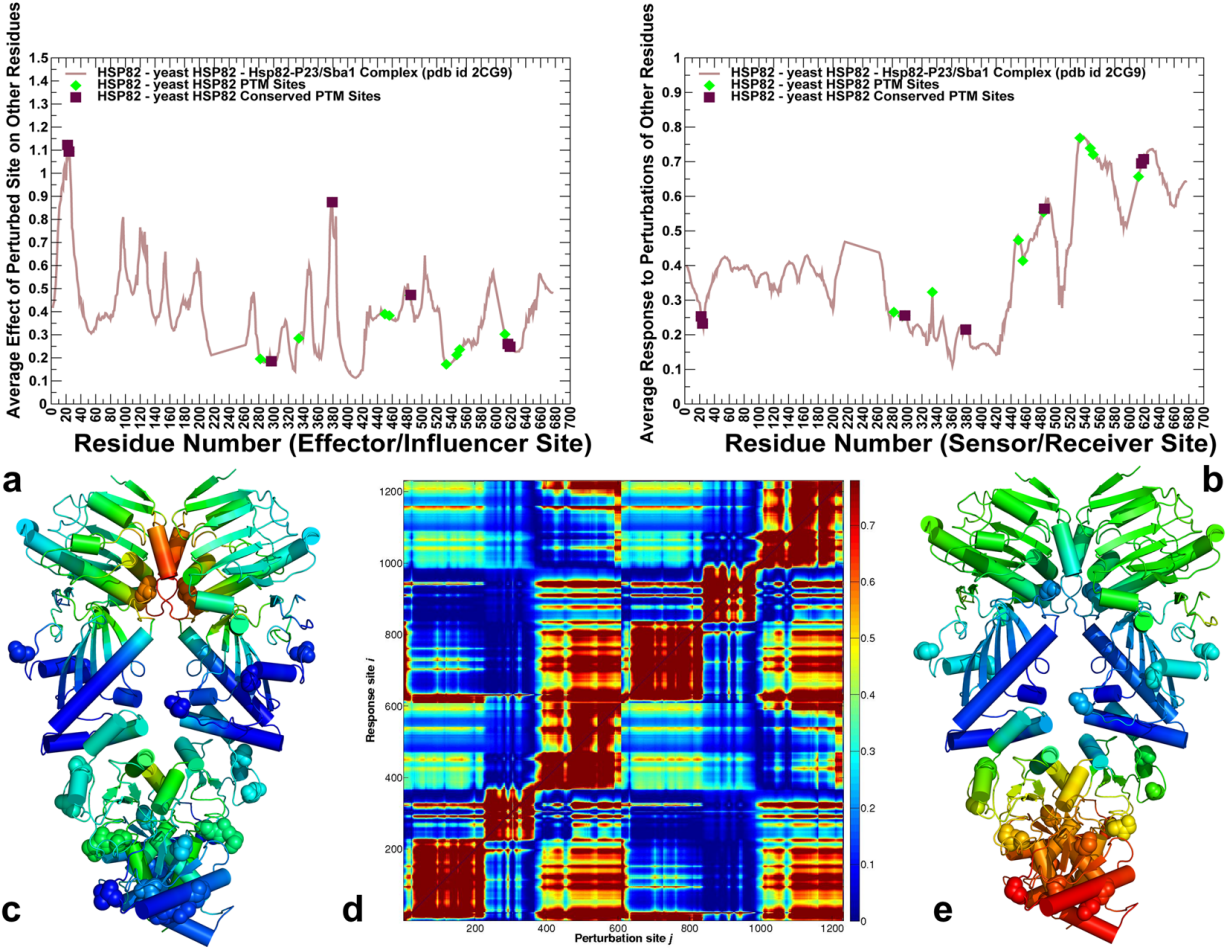
The PRS Analysis of the yeast Hsp82 Identifies Effector and Sensor Sites of Allosteric Signaling. (**a**) The residue-based effector profile measures the average impact of the perturbed site on all other residues in the Hsp82 dimer structure. The effector profile value for a given residue is the average over all elements of the PRS matrix in the corresponding row. This parameter quantifies the average residue propensity to transmit perturbation. (**b**) The residue-based sensor profile measures the average response of a given residue to perturbations of all other residues in the Hsp82 dimer. The sensor profile value for a given residue is the average over all elements of the PRS matrix in the corresponding column. This metric estimates the average residue propensity to sense perturbations of other residues. In panels (a,b) the effector/sensor parameter values for PTM sites are highlighted as green filled diamonds, and for conserved PTM sites in filled maroon squares. (**c**) The crystal structure is color-coded by ability to propagate perturbations, where red regions are strongest effectors. (**d**) The normalized PRS heat map where the strongest signals of high sensor/effector residues are shown in dark red. (**e**) The crystal structure of yeast Hsp82 is color-coded by sensitivity to perturbation. Red/orange regions are the most susceptible sensor sites, while dark-blue regions are the most insensitive sites; green/cyan regions show moderate sensitivity to perturbations.

By examining the PRS profiles in yeast Hsp82 structures (Fig. 7a,b), we found that conserved PTMs often corresponded to effector residues, while the majority of flexible PTM sites were featured among sensor centers. Indeed, conserved PTM sites in the NTD (T22, Y24) and MD regions (S379) matched precisely with the sharp dominant peaks of the distribution (Fig. 7a,c). Accordingly, these three residues can form global mediating centers that control propagation of allosteric communications in the Hsp82 dimer. It is well established that T22 and Y24 sites contribute to stabilization of the inter-monomer interface in the closed Hsp82 dimer^36^. According to our results, these residues can exert both local and global effects on chaperone activity through their role as major effectors of signal transmission. Similar arguments could reconcile strong effects of phosphorylation-mimicking S379 variants on the rates of ATP hydrolysis^38^. A close proximity of S379 to the ATP binding site suggested that phosphorylation at this position may disrupt local structural environment in the catalytic loop and affect stability of the closed Hsp90 dimer^38^. Our results showed that S379 residue could also serve as a key mediator of long-range interaction governing signal transmission across the NTD-MD interface (Fig. 7a,c). While effector residues resided in the Hsp90-NTD and interfacial regions, sensor residues were consolidated at the exposed CTD regions that are enriched by PTM sites (Fig. 7b). Noteworthy, conserved PTM site S485 corresponded to a peak in the sensor profile (Fig. 7b,e), showing that this residue can be allosterically linked with the effector residue S379 in propagating global conformational changes. The allosteric coupling between these PTM sites can be critical for the inter-domain communications, explaining why phosphorylation-mimicking mutations at these positions can alter conformational equilibrium and impair progression of the ATPase cycle. Previous studies argued that differences in the local structural environments of S379 and S485 may lead to distinct mechanisms responsible for functional role of these PTM sites^38^. Our analysis suggested that these PTMs can be linked through a mechanism of allosteric coupling in which these sites play complementary roles as effector and sensor of signal transmission.

The PRS heat map highlighted the strength of the residue responses to perturbations in the Hsp82 dimer (Fig. 7d). The increased intensity red bright spots in the heat map indicate peak residues that experience large displacements, while blue-colored areas imply residues that experience only small displacement. Analysis of the PRS matrix showed that perturbations can exert both local effects and long-range changes that are manifested by the appearance of dark-red regions away from the diagonal (Fig. 7d). The PRS heat map matrix illustrated a significant density of the allosterically important residues (mostly effectors) in the Hsp82-NTD and inter-domain regions. A group of Hsp82-MD residues (F364, F421, E431) that are involved in the inter-domain interactions and form mechanical hinge also featured high allosteric propensities in the PRS map. These results are consistent with the recent experimental analysis of regulatory points that control allosteric conformational changes in the Hsp82 chaperone^80^.

**Figure 8:**
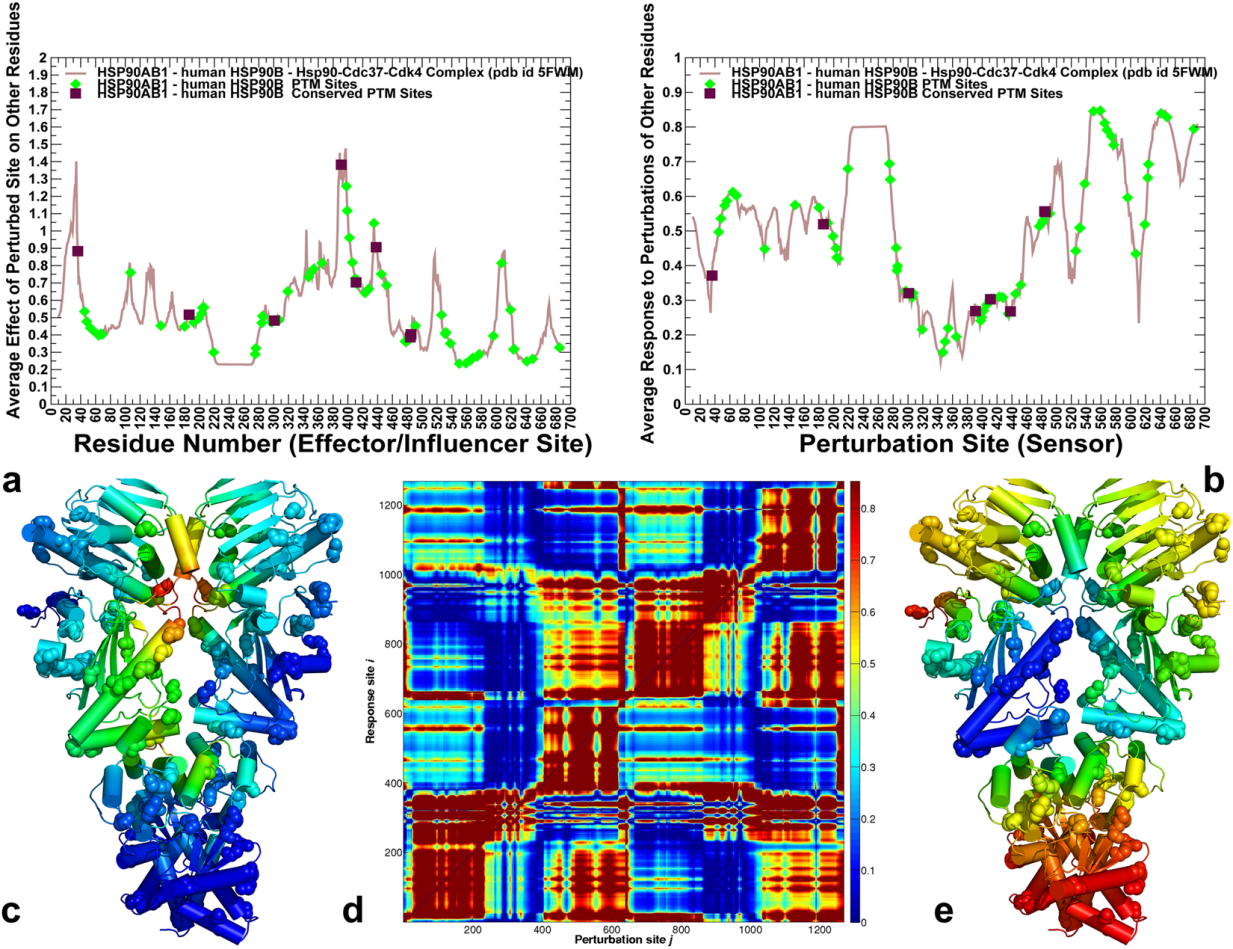
The PRS Analysis of the human Hsp90β Identifies Effector and Sensor Sites of Allosteric Signaling. (**a**) The residue-based effector profile measures the average impact of the perturbed site on all other residues in the human Hsp90β dimer structure. The effector profile value for a given residue is the average over all elements of the PRS matrix in the corresponding row. (**b**) The residue-based sensor profile measures the average response of a given residue to perturbations of all other residues in the human Hsp90β dimer. The sensor profile value for a given residue is the average over all elements of the PRS matrix in the corresponding column. In panels (a,b) the effector/sensor parameter values for PTM sites are highlighted as green filled diamonds, and for conserved PTM sites in filled maroon squares. (**c**) The crystal structure is color-coded by ability to propagate perturbations, where red regions are strongest effectors. (**d**) The normalized PRS heat map where the strongest signals of high sensor/effector residues are shown in dark red. (**e**) The crystal structure of the human Hsp90β is color-coded by sensitivity to perturbation. Red/orange regions are the most susceptible sites, called sensors, while dark-blue regions are the most insensitive sites; green/cyan regions show moderate sensitivity to perturbations.

A similar picture emerged from the PRS analysis of the human Hsp90β dimer (Fig. 8a,b). The peaks of the effector profile (Fig. 8a,c) corresponded to the nucleotide binding pocket residues in the NTD (T31, Y33, E42, L43) and catalytic loop in the Hsp90-MD (residues 382–402) with a particularly strong spike at S391 and a critical catalytic residue R392 (R380 in yeast Hsp82) that coordinates the γ-phosphate of ATP. Another peak corresponded to a hydrophobic cluster at the NTD-MD interface could be formed by interactions of conserved residues W289, P287, F304, Y356 and F208. This interaction cluster is conserved in the Hsp90 proteins^81^. Hence, the key effector residues in the Hsp90 dimer are assembled near the nucleotide binding site and the NTD-MD interface (Fig. 8a,c). Several PTM positions (K286, K350, S365 and K411) reside near the peaks of the effector profile (Fig. 8a), which is consistent with the roles of these sites as hinge centers and regulators of signal transmission.

The PRS heat map matrix similarly highlighted that effectors were located in the dimerization and inter-domain regions of the NTD and CTD (Fig. 8d). Our findings that yeast Hsp82 PTM residues (T22, Y24, S379) and human Hsp90β PTMs (T31, Y33) act as strong effector residues are consistent with recent computational study of human Hsp90α showing that corresponding Hsp90α positions correspond to allosteric hotspots^72^. Several other regions in human Hsp90β that corresponded to the effector profile peaks (E42, L43, and D382-K402) were also singled out as potential controllers of allosteric signaling in the analysis of human Hsp90α (E47, L48, and D390-K410 respectively)^72^. While many conserved PTM sites dominated the effector profile, less conserved PTM positions corresponded to the peaks of the sensor profile (Fig. 8b,e). A large fraction of PTM sites in CTD regions (K550, K559, K564, K568, K574 and K577) formed a major peak in the sensor profile, showing that these accessible PTM sites can act as major signal receivers and carriers of conformational changes (Fig. 8b,e). A broad agreement between our results and computational analysis of human Hsp90α chaperone^72^ also extends to the reported sensor profiles, highlighting conservation of effector and receiver hotspots in the Hsp90 proteins.

We also explored PRS analysis in the partially open and closed Grp94 forms to identify effector residues whose perturbations would enact functional conformational change (see Supplementary Figs S7, S8). The PRS profiles in the open Grp94 structure showed clear differences as effector centers were consolidated in the stable MD-CTD regions, while the majority of sensor sites occupied mobile NTD regions that undergo structural changes (see Supplementary Fig. S7). In contrast, in the closed Grp94 form the characteristic effector peaks resided in the NTD regions (see Supplementary Fig. S8), including the pre-NTD motif (residues 63–72), helix-1 (residues 81–92), and elements of the ATP-lid (residues 161–171). These findings are in accordance with the recent structural data showing that a region of the pre-N domain is required to regulate the ATPase rate and Grp94 dimer closure^51^. Similar observations in the analysis human Hsp90α suggested that nucleotide binding allosterically activates the closing transition by triggering the uncoupling of the ß-strand-1 and helix-1 from the ATP lid^72^. Our analysis also revealed presence of dominant effector peaks in the MD-CTD and CTD regions of Grp94, including residue clusters 494-LGVIED-499, 425-MMPKYL-430, and 658-MERIM-662 (see Supplementary Fig. S8). The reported sensor regions in the Grp94 closed dimer included a number of mobile PTM sites (K161, T165, K168) in the NTD that may serve as major receivers of allosteric signals enabling conformational transitions towards the partially open “v-like” conformation.

In summary, PRS analysis suggested that functional cross-talk between PTM residues in the Hsp90 proteins may be linked with their specific roles as effectors and sensors of allosteric signaling. In the proposed model of allosteric signaling, local perturbations of regulatory PTM sites in the NTD and NTD-MD regions would produce strong effector signals, while moderately conserved PTMs are largely localized in the CTD regions and act as strong sensors of perturbations in the system and major receivers of allosteric signal. We argue that this model may underlie the mechanistic basis of long-range communication between spatially separated binding sites in the Hsp90 proteins.

### Allosteric Communication Pathways in the Hsp90 Proteins Highlight Functional Roles of PTM Sites as Regulatory Switches

Using a graph-based representation of protein structures^82,83^, we integrated topological connectivity of protein residues and dynamic contact maps of residue cross-correlations^84^ with coevolutionary residue dependencies in the construction of the residue interaction network^46,85–87^. The network edges between nodes (residues) are weighted based on the MD-derived residue-residue couplings and coevolutionary residue correlations. In this model, allosteric communications are determined by short inter-residue paths that transmit signals through dynamically correlated and coevolutionary-coupled nodes. We explored edge betweenness (or edge centrality) in the global interaction network as a proxy for modeling of allosteric communication pathways. This parameter is defined as the ratio of all the shortest paths passing through a particular edge to the total number of shortest paths in the network. The central objective of this analysis was to quantify how allosteric signals may be transmitted between major effector and receiver centers localized in the NTD and CTD regions respectively.

**Figure 9:**
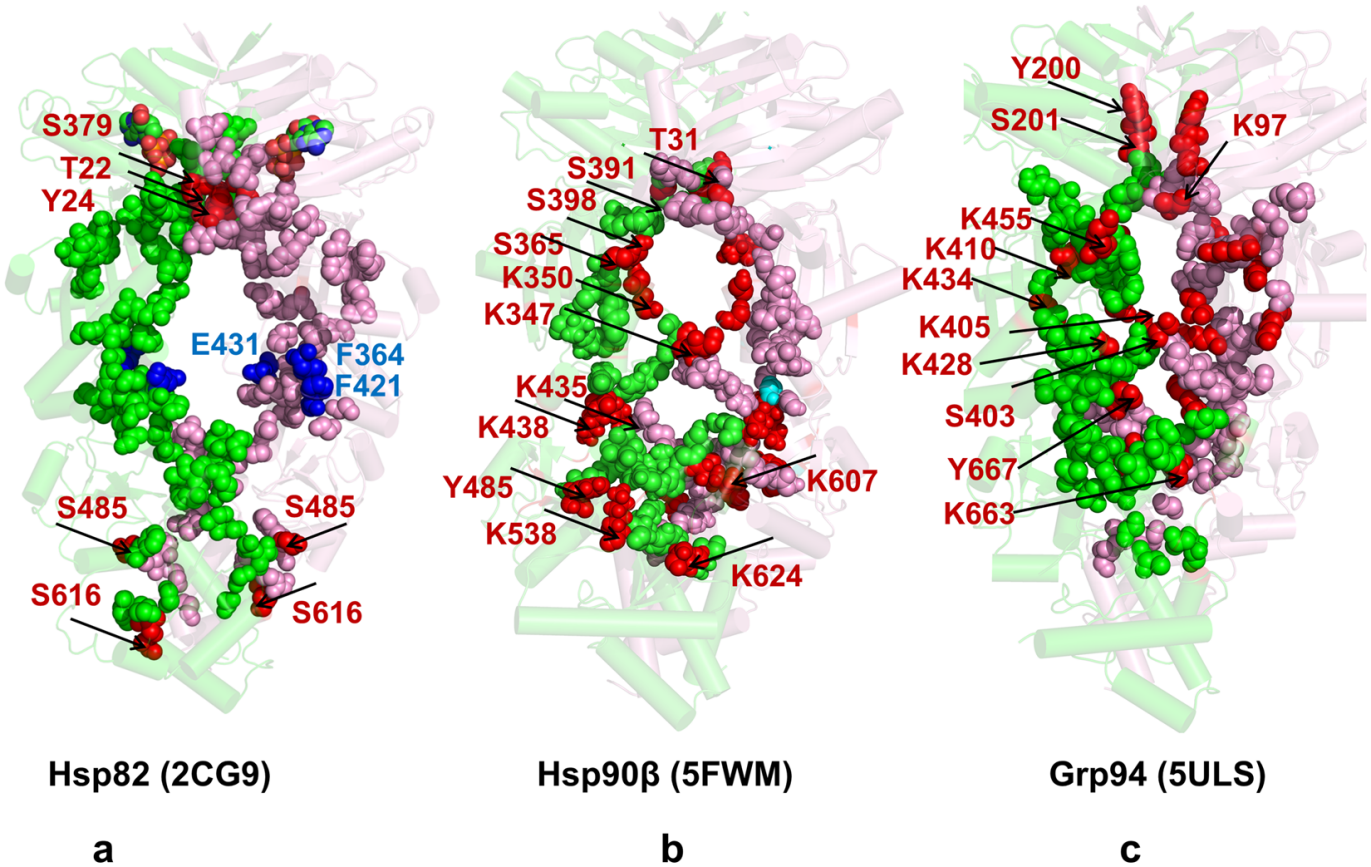
Structural Maps of Allosteric Communication Pathways between Major Effector and Sensor Centers in the Hsp90 Proteins. Structural map of major communication pathways connecting major effector and sensor centers through high centrality edges in the closed dimer forms of yeast Hsp82 (**a**), human Hsp90β (**b**), and canine Grp94 (**c**). The Hsp90 structures are shown in ribbons with reduced transparency to enhance clarity of communication paths. The monomer A is shown in green ribbons and monomer B in pink ribbons. The depicted pathways represent most probable routes that connect effector centers in the Hsp90-NTD with sensor sites in the Hsp90-CTD regions. Residues along these pathways are shown in green and pink spheres for respective monomers. The positions of PTM sites in the Hsp90 proteins are highlighted by red spheres and annotated. Note that conserved PTM sites can often assume role of high centrality bridges forming hubs in communication pathways. The position of experimentally known regulatory control points in the yeast Hsp82 chaperone^80^ are shown on blue spheres and annotated.

Analysis of high centrality bridges in the yeast Hsp82 revealed a number of mediating bridges that may be important for long-range signal propagation (Fig. 9a). We observed that Y22, T24 and S379 effector residues are interconnected and form a dense hub in the Hsp82-NTD that can coordinate signal transmission from the ATP binding site to the interdomain regions and CTD binding site. These observations are consistent with PRS analysis revealing that S379 has strong propensities for mediating allosteric interactions and controlling signal transmission across the NTD-MD interfaces (Fig. 7a,c). Atomistic reconstruction of communication pathways between effector and sensor sites unveiled topography and composition of short routes connecting NTD and CTD regions, confirming that S379 and S485 PTM sites can serve as gate-keepers of signal transmission in the Hsp82 dimer (Fig. 9a). Importantly, optimal short paths linking major effector and sensor centers in the NTD and CTD regions would navigate through allosteric control points in the Hsp82-MD (F364, F421, E431)^80^. These findings may provide an additional insight in rationalizing deleterious mutational effects of these residues on the ATPase activity as perturbations in these regulatory control points may reduce the fidelity and efficiency of allosteric signaling in the Hsp82 dimer. Based on atomistic models of signaling paths in the Hsp82 dimer, we argue that a cross-talk between effector PTM sites (T22, Y24, S379) and sensor PTM sites (S485, S616,S619) can be instrumental in modulation of allosteric signaling and ATPase activity. In this mechanism, single control points T22, T24, S379, and S485 can act as regulatory switches of conformational equilibrium and client interactions during the Hsp90-ATPase cycle.

Structural models of communication paths in the human Hsp90β connecting major effector and sensor sites also revealed a considerable involvement of phosphorylation and lysine acetylation sites (Fig. 9b). Our findings showed that a number of conserved PTM sites (T31, Y33, S365, S391, S398, K399, K411, and K350) can contribute to optimal signaling routes. Some of these PTM sites are interconnected (K350-S365) and can form allosteric hubs along communication pathways. These observations may be important in light of recent data revealing that S365 is an isoform-specific phosphorylation site acting as a regulatory switch of the conformational equilibrium in the Hsp90β chaperone^39^. The critical role of this PTM site in signal propagation may explain why mutations in this position may divert the progression of the Hsp90 cycle and impair kinase client binding^39,88^.

A communication map in the closed Grp94 dimer similarly indicated that PTM sites can be involved in mediating long-range signaling (Fig. 9c). Allosteric communications in Grp94 may proceed via interaction bridges that engage several PTM sites: Y94-S447, G402-S403, S403-K404, K404-D421, Q452-K455, K473-D593 (Fig. 9c). According to our results, S403, S447 (of the catalytic 447-SRE-449 motif) and K404 can serve as important hubs of allosteric signaling in the Grp94 dimer. Another group of high centrality bridges (K410-Y412, K410-G435, and G435-K4340) involved conserved PTM sites K410 and K434 that mediate inter-domain couplings. Hence, efficiency of global communication traffic in the Hsp90 dimers may critically depend on the interaction bridges mediated by PTM sites. Our results suggested that allosteric communications between effector and sensor regions in the Hsp90 dimer may be enabled through efficient network connectivity between PTM sites. This network-centric mechanism may be operational during synchronization multiple PTMs through a combinatorial code in the proteome context^26^. By reconstructing the network of short paths transducing conformational information in the Hsp90 proteins, we suggest that allosteric coupling between PTMs could provide additional layers for regulation of chaperone activity and binding of client proteins.

## Discussion

Previous studies reported that cross-talk events preferentially occur among nearby PTM sites and cross-talking pairs tended to coevolve^7^. Another study suggested that coevolutionary dependencies between some PTM sites may bias their structural proximity, masking a more global statistical tendency of all PTMs to be spatially separated and allow for simultaneous access to different sites^89^. By examining the interplay between coevolution, dynamics and allosteric communications in the Hsp90 structures, this study provided new insights into mechanisms underlying functional coupling and cross-talk between PTM sites. We determined that PTM sites occupy critical positions in slow modes, where a small fraction of conserved PTMs form spatially separated regulatory hinge centers, while the majority of coevolving flexible PTMs serve as integral nodes and form local clusters in cooperatively moving regions. These findings were further substantiated in the PRS analysis of allosteric residue responses to external perturbations. By employing a battery of complementary dynamic and network-based approaches, we presented consistent evidence that a small group of conserved PTM hotspots can play an important role in orchestrating allosteric signaling in the Hsp90 proteins, while less conserved and more flexible PTMs act as main transmitters of conformational changes. According to our results, functional cross-talk between PTM sites may be determined by their respective roles as effectors and sensors of allosteric signaling in the Hsp90 structures. The complementary roles of PTMs as effectors and sensors of allosteric signaling suggested how these sites may become involved either in positive or negative cross-talk^41,90,91^. In a positive cross-talk, a particular PTM serves as a signal for the addition or removal of a second PTM, while a negative crosstalk is often defined as direct competition between PTM sites where one modification masks the recognition site for a second PTM. We argue that plasticity of a combinatorial PTM core in the chaperone may be enacted through coupling between effector and sensor PTM sites, allowing for regulatory switching and rapid responses to structural requirements of multiple modified enzymes. The proposed computational framework that links dynamics and allosteric responses in multi-domain chaperone systems with diversity and functions of PTM sites may be useful in developing therapeutic strategies for designing robust combinations of targeted and allosteric inhibitors of chaperones and oncogenic kinase clients.

## Materials and Methods

### Atomistic Molecular Dynamics Simulations

All-atom 500 ns MD simulations have been performed for the crystal structure of ATP-bound yeast Hsp82 (pdb id 2CG9)^48^ (Fig. 1a), the cryo-electron microscopy (EM) structure of ATP-bound human Hsp90β (pdb id 5FWM)^49^ (Fig. 1b), and crystal structures of ATP-bound forms of canine Grp94 (pdb id 2O1U, 5ULS)^50,51^ (Fig. 1c). All structures were obtained from the Protein Data Bank^92^. Structure preparation process included several main steps previously reported in detail in our studies of molecular chaperones^93–95^. In brief, in this protocol, hydrogen atoms and missing residues were initially assigned using the WHATIF program^96^. The unresolved structural segments in the crystal structures were modeled using MODELLER program^97^ program. We then employed template-based loop prediction approaches ModLoop^98^ and the ArchPRED^99^ to reconstruct and refine the linker region in the yeast Hsp82 and human Hsp90β structures, as well as Src-loop residues F329-D339 in chain A, and A597-K611 in both protomers of the yeast Hsp82structure. The protonation states on protein residues were generated with the WHATIF program and refined by the *H*++ web server^100^. MD simulations were performed using CHARMM22 force field^101^ and the explicit TIP3P water model^102^ in the NAMD 2.6 package^103^.

### Discrete Molecular Dynamics Simulations

We also employed the formalism of the discrete molecular dynamics (DMD) simulations^61–63^ to simulate Hsp90 structures (Fig. 1). In the DMD approach, the protein structures were modeled as systems consisting of *C*_*α*_ residue-based beads interacting through a discontinuous square well potential. In the basic DMD formalism^61^ particles move in the ballistic regime under constant velocity until a collision between a pair of particles occurs at the distance where their pairwise potential energy changes, i.e. DMD consists of a sequence of atomic collisions. In the absence of any collision, the particles move linearly with constant velocity. The main advantage of DMD is elimination of time-consuming computations of forces and accelerations as compared to more demanding atomistic molecular dynamics MDs. In the DMD implementation, the interaction potentials are defined as infinite square wells, such that the particle-particle distances vary between ${d}_{min}=(1-\sigma ){r}_{ij}^{0}$ and ${d}_{min}=(1+\sigma ){r}_{ij}^{0}$ where ${r}_{ij}^{0}$ is the distance between particles (residues) *i* and *j* in the native conformation and 2*σ* is the width of the square well. The MD-averaged conformation was taken as the native conformation. Residue-residue interaction potentials are defined for the particles at a distance smaller than a cut-off radius *r*_*c*_ in the native conformation. A small well width *σ* = 0.05 was used for neighboring particles to keep the *C*_*α*_ − *C*_*α*_ distances closer to the expected equilibrium value of 3.8 Å. For nonconsecutive pairs of *C*_*α*_ particles, *r*_*c*_ = 8 Ǻ and *σ* =  0.1 were used. Using DMD simulations, we generated conformational landscapes of the Hsp90 proteins in a coarse-grained representation. The coarse-grained conformational ensembles of the Hsp90 structures were then subjected to all-atom reconstruction using PULCHRA method^64^ and CG2AA tool^65^ that derived atomistic structures from simulation trajectories. The all-atom conformations were additionally optimized using the 3Drefine method^66^ that utilizes atomic-level energy minimization with a composite physics and knowledge-based force fields.

### Mutual Information Networks of Coevolving Protein Residues

Analysis of coevolving residues was carried out using mutual information (MI) between two positions in the multiple sequence alignment (MSA), which reflects the extent to which knowing the amino acid at one position can predict the amino acid identity at the other position. MI was calculated between pairs of columns in the MSA using MISTIC approach^46,85,86^. MI is a nonlinear statistic that measures the information between two random and discrete variables. Given that any two positions in the MSA can be considered random variables *x* and *y*, the MI between is these two positions are given by the relationship:

1 $$MI(i,j)={\sum }_{{a}_{i},{b}_{j}}P({a}_{i},{b}_{j})\ast \mathrm{log}(\frac{P({a}_{i},{b}_{j})}{P({a}_{i})\ast P({b}_{j})})$$

$P({a}_{i},{b}_{j})$ is the frequency of amino acid $a$ occurring at position *i* and amino acid *b* at position *j* of the same sequence. *P*(*a*_*i*_) is the frequency of amino acid *a* occurring at position *i* and *P*(*b*_*j*_) is the frequency of amino acid *b* at position *j*. The amino acid frequency pair $P({a}_{i},{b}_{j})$ is calculated as $N({a}_{i},{b}_{j})/N\,$ where $N({a}_{i},{b}_{j})$ is the number of times that an amino acid pair $({a}_{i},{b}_{j})$ is observed at positions *i* and *j* respectively. *N* is the total number of sequences in the sequence alignment. MSA were obtained from Pfam^47^, a database of protein families that includes their annotations and MSA information generated using hidden Markov models. The seed alignments of the HATPase_c family (PF02518) and Hsp90 family (PF00183) were used as the curated alignment which contains representative sequences of the chaperone family, from which a profile hidden Markov model (HMM) is generated using the HMMER3 program (http://hmmer.janelia.org/). Each profile is then searched against a primary sequence database based on UniProtKB to create the full MSA profile. The criteria parameters for generating a MSA are the E-value and the number of columns in the multiple-sequence alignment for which sufficient sequences can be found to infer evolutionary couplings. Based on these criteria, for sequences in the dataset, an E-value of 10^−2^ and a column-inclusion threshold of 80% were used in the MSA generation. All sequences in the full MSA score above curated thresholds are included in the full alignments. The Kullback-Leibler (KL) divergence score $KLConsScore$ was calculated in the framework of MISTIC approach^46,85,86^ using a combined MSA profile generated in the Pfam database^47^ for the HATPase_c family, (PF02518, Hsp90-NTD regions) and the Hsp90 family (PF00183, Hsp90-MD and Hsp90-CTD regions). The KL parameter measures the population diversity of residues at a single MSA position and takes into account biochemical environment by considering frequency of mutations at each position with respect to the background amino acid frequencies calculated from the MSA. For each column of the MSA, the KL conservation is calculated according to the following formula:

2 $$KLConsScor{e}_{i}={\sum }_{i=1}^{N}ln\frac{P(i)}{Q(i)}$$

Here, *P*(*i*) is the frequency of amino acid *i* in that position and *Q*(*i*) is the background frequency of the amino acid in nature calculated using an amino acids background frequency distribution. In the coevolutionary analysis, we computed the following parameters: a cumulative mutual information score $cMI$, and the proximity mutual information score $pMI$. The parameter $cMI\,$is a mutual information score per residue position that characterizes the extent of mutual information in its physical neighborhood. This sequence-based parameter defines an extent to which a given amino acid residue contributes to a mutual information network. The parameter $cMI$ is calculated as the sum of MI values above a certain threshold (MI > 6.5) for every amino acid pair in which a particular residue of interest appears. The MI threshold of 6.5 was shown to be an adequate and reliable lower boundary of mutual information interactions^46,85,86^.

3 $$cM{I}_{x}={\sum }_{y,MI(x,y) > t}M{I}_{(x,y)}$$

$pMI$ score for each position is defined as the average of $cMI\,$scores of all the residues within a certain physical distance from a given residue when mapped on the protein kinase structure. The distance between each pair of residues in the structure was calculated as the shortest distance between any two atoms, other than hydrogen atoms, that belong to each of the two positions. The threshold d of 5 Å defines structural proximity of each residue in defining $pMI$ score^46^:

4 $$pM{I}_{x}=\frac{1}{N}{\sum }_{d(x,y) < t}cM{I}_{(x,y)}$$

### Perturbation Response Scanning

Perturbation Response Scanning (PRS) approach^73–75^ is based on the linear response theory and allows to evaluate residue displacements in response to external forces. In this approach, it is assumed that the 3*N* × 3*N* Hessian matrix, ${\boldsymbol{H}}$, whose elements represent second derivatives of the potential at the local minimum connect the perturbation forces to the residue displacements. A perturbation force is applied to one residue at a time, and the response of the protein system is measured by the displacement vector ${\rm{\Delta }}{\boldsymbol{R}}(i)={{\boldsymbol{H}}}^{-1}{{\boldsymbol{F}}}^{({\boldsymbol{i}})}$ that is then translated into *N* × *N* PRS matrix. The second derivatives matrix ${\boldsymbol{H}}$ can be obtained from DMD simulation trajectories of each structure, with residues represented by C_α_ atoms and the deviation of each residue from an average structure was calculated by $\,{\rm{\Delta }}{{\bf{R}}}_{i}(t)=\,{{\bf{R}}}_{i}(t)-\,\langle {{\bf{R}}}_{i}(t)\rangle$, and corresponding covariance matrix C was then calculated by $\,{\rm{\Delta }}{\bf{R}}{\rm{\Delta }}{{\bf{R}}}^{T}$. We sequentially perturbed each residue in the respective Hsp90 structures by applying a total of 250 random forces to each residue to mimic a sphere of randomly selected directions as suggested in^78^. The displacement changes, ${\rm{\Delta }}{{\boldsymbol{R}}}^{{\boldsymbol{i}}}$ is a *3N-*dimensional vector describing the linear response of the protein and deformation of all the residues. Using the residue displacements upon multiple external force perturbations, we compute the magnitude of the response of residue *k* as $\langle {|\Delta {{\boldsymbol{R}}}_{{\boldsymbol{k}}}^{({\boldsymbol{i}})}|}^{2}\rangle$ averaged over multiple perturbation forces ${{\boldsymbol{F}}}^{i}$ yielding the *ik*^th^ element of the *N* × *N* PRS matrix^76–78^. The average perturbation response maps of the protein are matrices in which the *ij*th element refers to the average response (displacement) of residue $j$ to external perturbation at residue $i$.

The average effect of the perturbed effector site *i* on all other residues is computed by averaging over all sensors (receivers) residues $j$ and can be expressed as ${\langle {(\Delta {{\boldsymbol{R}}}^{{\boldsymbol{i}}})}^{2}\rangle }_{effector}$. In turn, the *j*^th^ column of the PRS matrix describes the sensitivity profile of sensor residue $j$ in response to perturbations of all residues and is denoted as ${\langle {({\rm{\Delta }}{{\boldsymbol{R}}}^{{\boldsymbol{i}}})}^{2}\rangle }_{sensor}$. The sensor profile measures the ability of residue *j* to serve as a receiver (or transmitter) of dynamic changes in the system. Accordingly, the maxima along the sensor profile correspond to functional residues in cooperatively moving regions involved in execution of allosteric structural changes.

### Residue Interaction Networks and Modeling of Allosteric Communication Pathways

A graph-based model of protein structure considers residues as network nodes while inter-residue edges represent residue interactions. The details of graph construction using residue ${I}_{min}$ cutoff^82,83^ were outlined in our previous studies of the Hsp90 and Hsp70 chaperones^87,93–95^. The edges in the residue interaction network are weighted based on dynamic residue correlations couplings obtained from MD simulations^84^ and coevolutionary mutual information^85–87^. In this model, weight ${w}_{ij}$ is defined as the element of a matrix measuring the generalized correlation coefficient ${{\boldsymbol{R}}}_{MI}({{\boldsymbol{X}}}_{{\boldsymbol{i}}},{{\boldsymbol{X}}}_{{\boldsymbol{j}}})$ between residue fluctuations in structural and coevolutionary dimensions. The composite residue vector describes structural residue positions and respective proximity-based coevolutionary score^87^:

5 $${w}_{ij}=-\,\mathrm{log}[{{\boldsymbol{R}}}_{MI}({{\boldsymbol{X}}}_{{\boldsymbol{i}}},{{\boldsymbol{X}}}_{{\boldsymbol{j}}})]$$

The edge lengths in the network are thus obtained using the generalized correlation coefficients ${{\boldsymbol{R}}}_{MI}({{\boldsymbol{X}}}_{{\boldsymbol{i}}},{{\boldsymbol{X}}}_{{\boldsymbol{j}}})\,$ associated with the dynamic correlation and mutual information shared by each pair of residues. The length (i.e. weight) ${w}_{ij}=-\,\mathrm{log}[{{\boldsymbol{R}}}_{MI}({{\boldsymbol{X}}}_{{\boldsymbol{i}}},{{\boldsymbol{X}}}_{{\boldsymbol{j}}})]$ of the edge that connects nodes *i* and *j* is calculated from the corresponding generalized correlation coefficient between these nodes^104^. The networks edges were constructed based on the generalized correlations between all residues obtained from multiple simulations. Network edges were weighted for residue pairs with ${{\boldsymbol{R}}}_{MI}({{\boldsymbol{X}}}_{{\boldsymbol{i}}},{{\boldsymbol{X}}}_{{\boldsymbol{j}}}) > 0.5$ in at least one independent simulation as was previously described^105^. The ensemble of shortest paths is determined from matrix of communication distances by the Floyd-Warshall algorithm^106^ that compares all possible paths between each pair of residue nodes.

At the first step, the distance between connected residues was considered to be one, and the shortest path was identified as the path in which the two distant residues were connected by the smallest number of intermediate residues. Network graph calculations were performed using the python package NetworkX^107^.

### Data Availability

All data generated or analyzed during this study are included in this published article (and its Supplementary Information files).

## Electronic supplementary material

Supplementary Information
