# Supplementary material for: Dissecting Structure-Encoded Determinants of Allosteric Cross-Talk between Post-Translational Modification Sites in the Hsp90 Chaperones
Source: Sci Rep. 2018 May 2;8:6899. doi: 10.1038/s41598-018-25329-4 (PMC5932063; doi:10.1038/s41598-018-25329-4)
Supplement: Supplementary file 1 — Supplementary Information [file 41598_2018_25329_MOESM1_ESM.pdf]

Supplementary Information

Dissecting Structure-Encoded Determinants of Allosteric Cross-Talk between Post-Translational Modification Sites in the Hsp90 Chaperones

Gabrielle Stetz[1], Amanda Tse[1], Gennady M. Verkhivker[1,2*]

[1]Department of Computational and Data Sciences, Schmid College of Science and Technology, Chapman University, Orange, California, United States of America,

[2]Chapman University School of Pharmacy, Irvine, California, United States of America

*corresponding author

E-mail: verkhivk@chapman.edu

Supplementary Note

Coevolutionary Analysis of the PTM Sites in the Hsp90 Proteins

Although structural stability and evolutionary conservation may represent important characteristics of the regulatory sites, many other residues, particularly located in the buried hydrophobic regions may exhibit similar properties. To determine features that can differentiate regulatory PTM sites from nonfunctional conserved residues we also investigated coevolution of the Hsp90 residues. Coevolution of protein residues is a result of compensatory amino acid substitutions in a protein sequence that take place when a residue change in one region is dependent on the substitution of a residue at another region. Using MISTIC approach[46,88,89] we characterized the extent of mutual information (MI) and coevolutionary dependencies between residue pairs in the Hsp90 proteins. In this method, the residue-based MI score characterizes the extent of mutual information shared by a given residue with all other protein residues across protein family. Based on MI scores and using MISTIC server[46], a network of coevolutionary residues was constructed where nodes are residues and links between nodes represent coevolutionary signal between residue pairs. To analyze the network of coevolving residues in the Hsp90 proteins, we computed cumulative mutual information (cMI) and the proximity mutual information residue score (pMI) (see Supplementary Fig. S6 online). cMI score is a sequence-based parameter that measures the degree of shared mutual information of a given residue with the other protein residues. pMI score encapsulates both sequence and structural variations, defined as the average of cMI scores of all the residues within a predefined distance from a given residue in the protein structure. Using DMD-based conformational ensembles, we expanded the original definition of the pMI score. For each residue, pMI score was computed using ensemble-based representation of local residue proximity that averaged the amount of

mutual information shared between a given residues and its spatially close neighbors over the course of simulation.

Analysis of cMI profiles revealed that a number of PTM sites in yeast Hsp82 corresponded to the highly coevolving residues (high cMI score) and these residues typically mapped on conformationally flexible regions (Supplementary Fig. S6 online). Structural mapping of high cMI residues showed a diverse coverage across all domains, pointing to a potential connectivity between these sites in providing communication routes in the Hsp82 dimer (see Supplementary Fig. S6 online). According to our results, the PTM sites associated with moving regions in global modes typically feature high cMI scores (S484, S485, S616, and S619) as these residues can serve as carriers of conformational changes and allosteric signals in the Hsp82 dimer. At the same time, S297 and S379 sites showed markedly larger pMI scores, indicating that these regulatory hinge centers may be surrounded by highly coevolving residues that execute allosteric changes and enable signal transmission. The cMI profile indicated that residues close in sequence to S297 and S379 featured high coevolutionary scores. Mapping of highly coevolving residues (top cMI score) and PTM sites onto the crystal structure of yeast Hsp82 (Supplementary Fig. S6 online) showed that these residues may often overlap and form local interaction clusters. In particular, dense clusters of high cMI residues could be seen in the close proximity of the regulatory PTM hotspots T22, Y24, and S379. Notably, high cMI residues are often only moderately conserved. A high degree of coevolution for the collectively moving residues can be a consequence of moderate sequence conservation that allowed for compensatory amino acid substitutions. We discovered that PTM sites in the NTD and MD regions featured moderate cMI score, while conserved PTMs in the CTD regions corresponded to highly coevolving positions. These results provided further explanation of the preferential

enrichment of PTM sites in the CTD regions, particularly residues 600–610 of the yeast Hsp82. A significant density of highly coevolving residues in the Hsp82-CTD is also consistent with the notion that moving residues involved in protein conformational changes are evolutionarily coupled with other sites to enable concerted dynamic rearrangements of residue interactions. The moderately conserved PTM sites forming local clusters in this region often correspond to high cMI residues, suggesting that spatial proximity and coevolutionary coupling of PTM sites may be leveraged to facilitate allosteric conformational changes in the Hsp82 dimer. We also found that PTM sites with top cMI scores are more likely to have links and form interactions with other high cMI residues. As a result, these PTM sites can mediate coevolutionary network that acts as a direct path for transmitting coevolutionary signals between remote mobile regions. We argue that to provide efficient control of communication pathways, moving PTM residues are clustered together in the Hsp82-CTD and are connected with many other coevolving sites in the protein structure.

On the other hand, PTM sites in the NTD regions and inter-domain interfaces are characterized by higher pMI values (Supplementary Fig. S6 online). Accordingly, regulatory hinge centers containing conserved PTM sites tend to be enriched by high pMI residues that control propagation of allosteric signal in the Hsp82 dimer. These results argue that a small fraction of conserved PTM sites with high pMI scores may play role of effectors in allosteric signaling. At the same time, a significant fraction of moderately conserved PTM sites that characterized by high cMI scores may function as sensors and major carriers of allosteric communications. These data suggest that flexible PTM residues involved in transmission of protein motions may be under different evolutionary constraints than other functional sites.

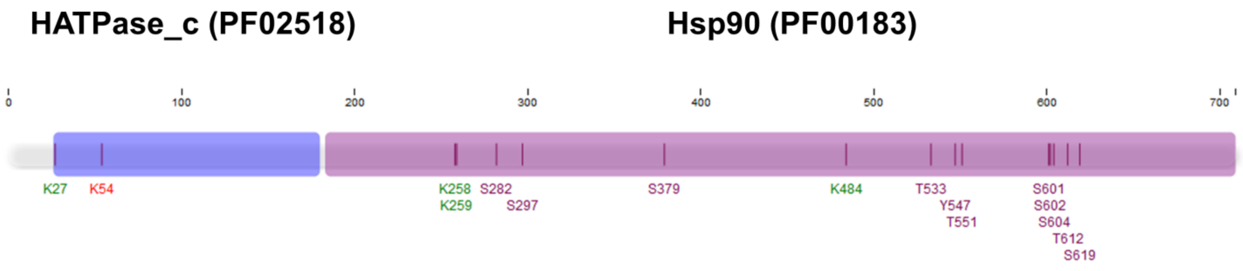

Supplementary Figure S1. The Distribution of PTM Sites in the yeast Hsp82 (UNIPROT HSP82_YEAST).

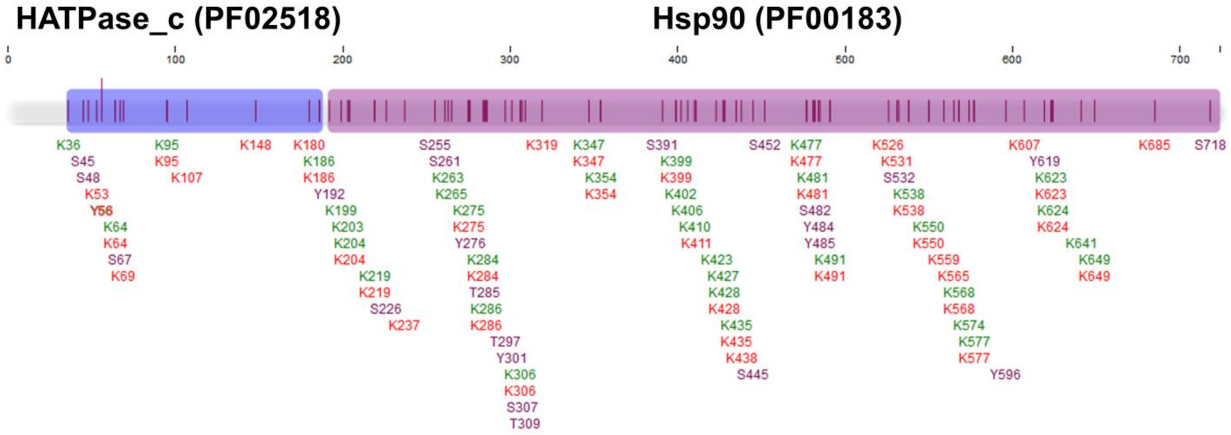

Supplementary Figure S2. The Distribution of PTM Sites in the human Hsp90β (UNIPROT HS90B_HUMAN)

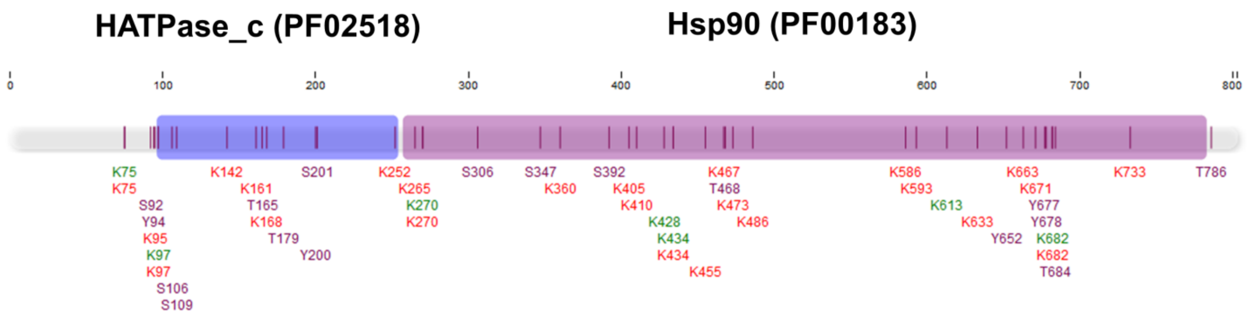

Supplementary Figure S3. The Distribution of PTM Sites in the canine Grp94 (UNIPROT ENPL_CANLF).

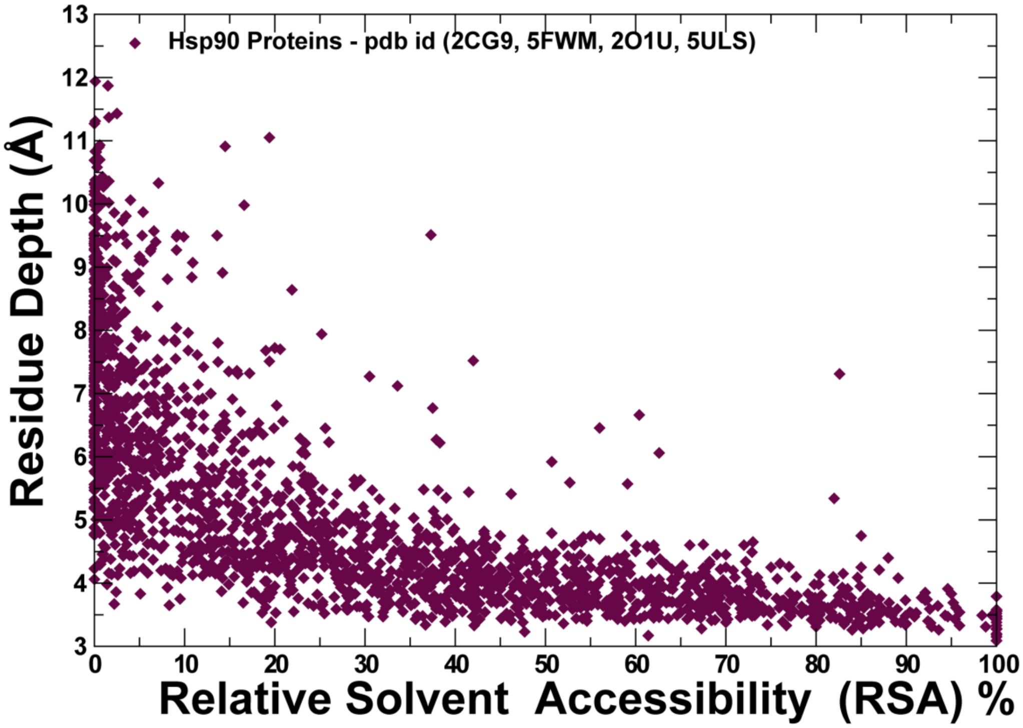

Supplementary Figure S4. The Scatter Distribution Plot between RSA and RD values for the Hsp90 Structures. The plots are generated by accumulating data points from average ensemble-averaged conformations for all studied Hsp90 structures. Note, when a residue is buried inside the protein structure, RD values are considerably more sensitive to the degree of residue burial as compared to RSA.

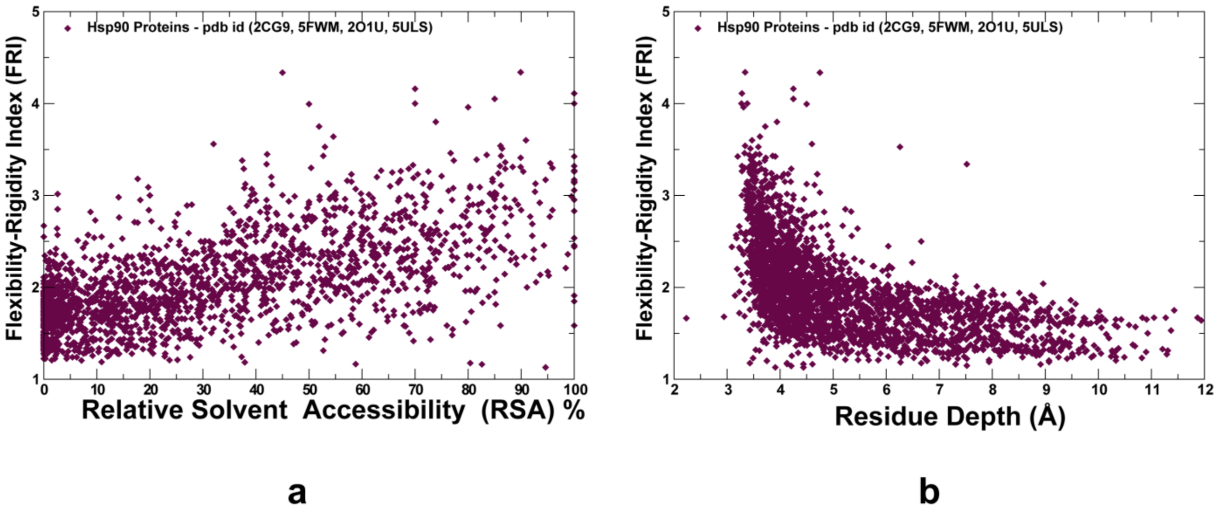

Supplementary Figure S5. The Relationship between Conformational Flexibility and Residue Exposure in the Hsp90 Structures. (a) The scatter distribution plot between FRI and RSA values. (b) The scatter distribution plot between FRI and RD values. The plots are generated by accumulating data points from ensemble-average conformations for all studied Hsp90 structures.

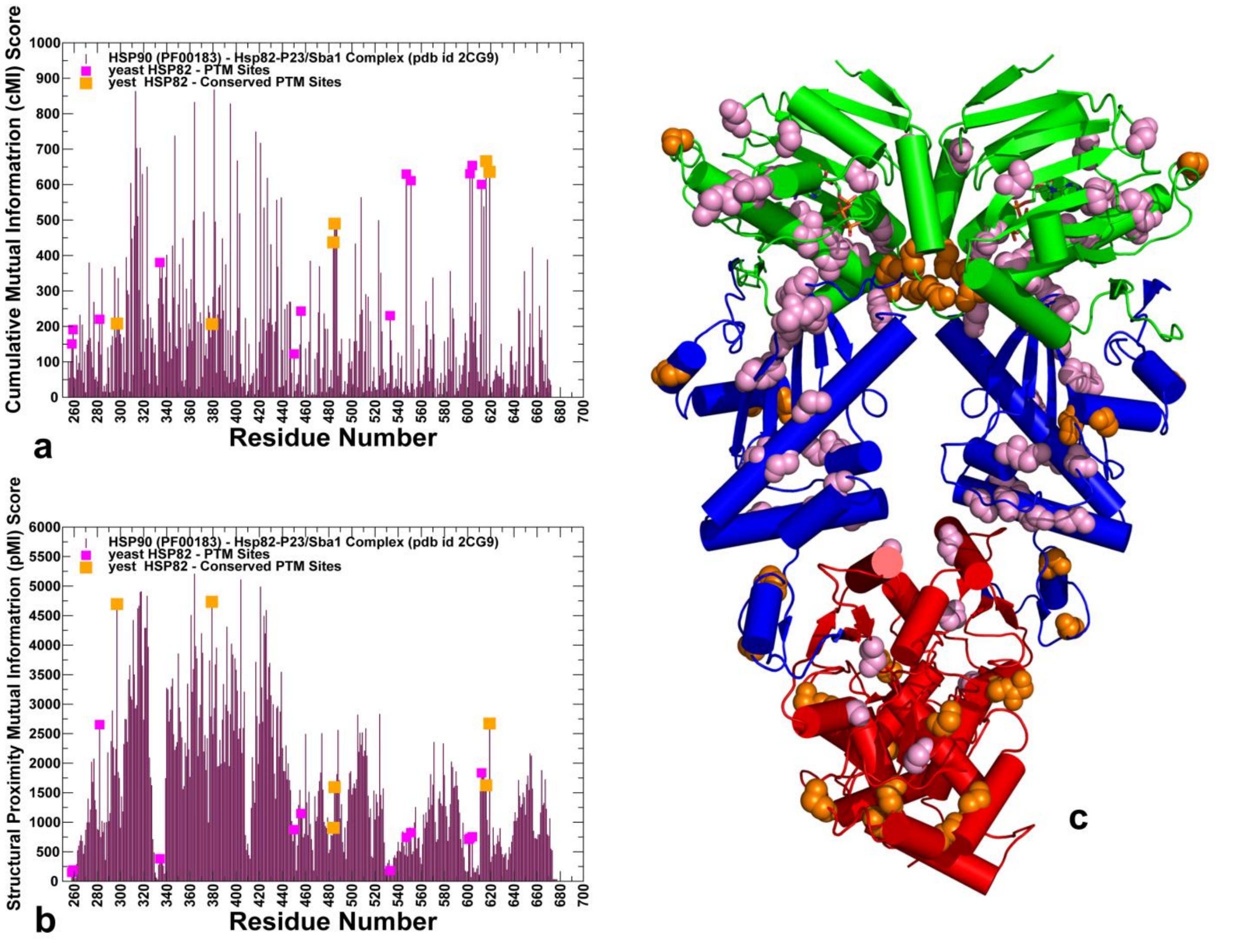

Supplementary Figure S6. Coevolutionary Analysis of the Hsp90 Proteins. (a) The residue-based cMI profile is mapped onto Hsp82 residues according to residue numbering in the crystal structure of yeast Hsp82 (pdb id 2CG9). (b) The residue-based pMI profile for the yeast Hsp82. The profiles are shown in maroon colored bars. The position of PTM sites is indicated by filled magenta squares and the conserved PTM sites (high KL score) are highlighted by filled orange squares. (c) Structural mapping of top 10% cMI residues (in pink spheres) and PTM sites in yeast Hsp82 (in orange spheres) onto the crystal structure of ATP-bound yeast Hsp82. The crystal structure of ATP-bound yeast Hsp82 (pdb id 2CG9) is shown in a ribbons and the domains are colored as follows: Hsp90-NTD (residues 2-216, in green), Hsp90-MD (residues 217-526, in blue) and Hsp90-CTD (residues 527-677, in red).

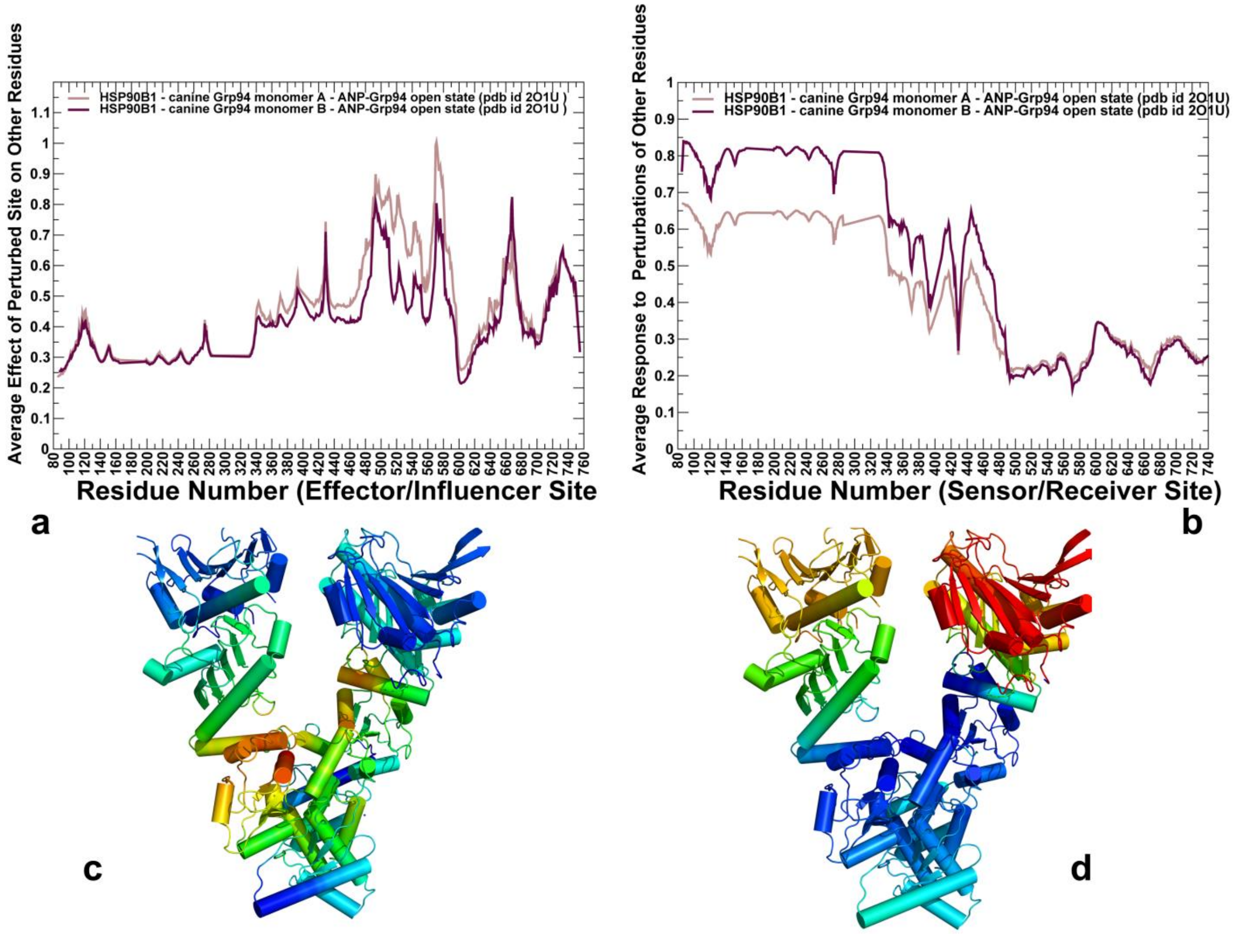

Supplementary Figure S7. The PRS Analysis of the Open Grp94 Dimer. (a) The residue-based effector profile measures the average impact of the perturbed site on all other residues in the partially open Grp94 dimer structure. (b) The residue-based sensor profile measures the average response of a given residue to perturbations of all other residues in the partially open Grp94 dimer. In panels (a,b) the effector/sensor profiles are shown for monomer A in light brown lines and for monomer B in maroon lines. (c) The crystal structure of the open Grp94 dimer form (pdb id 2O1U) is color-coded by ability to propagate perturbations, where red regions are strongest effectors. (d) The crystal structure is color-coded by sensitivity to perturbation. Red/orange regions are the most susceptible sensor sites, while dark-blue regions are the most insensitive sites; green/cyan regions show moderate sensitivity to perturbations.

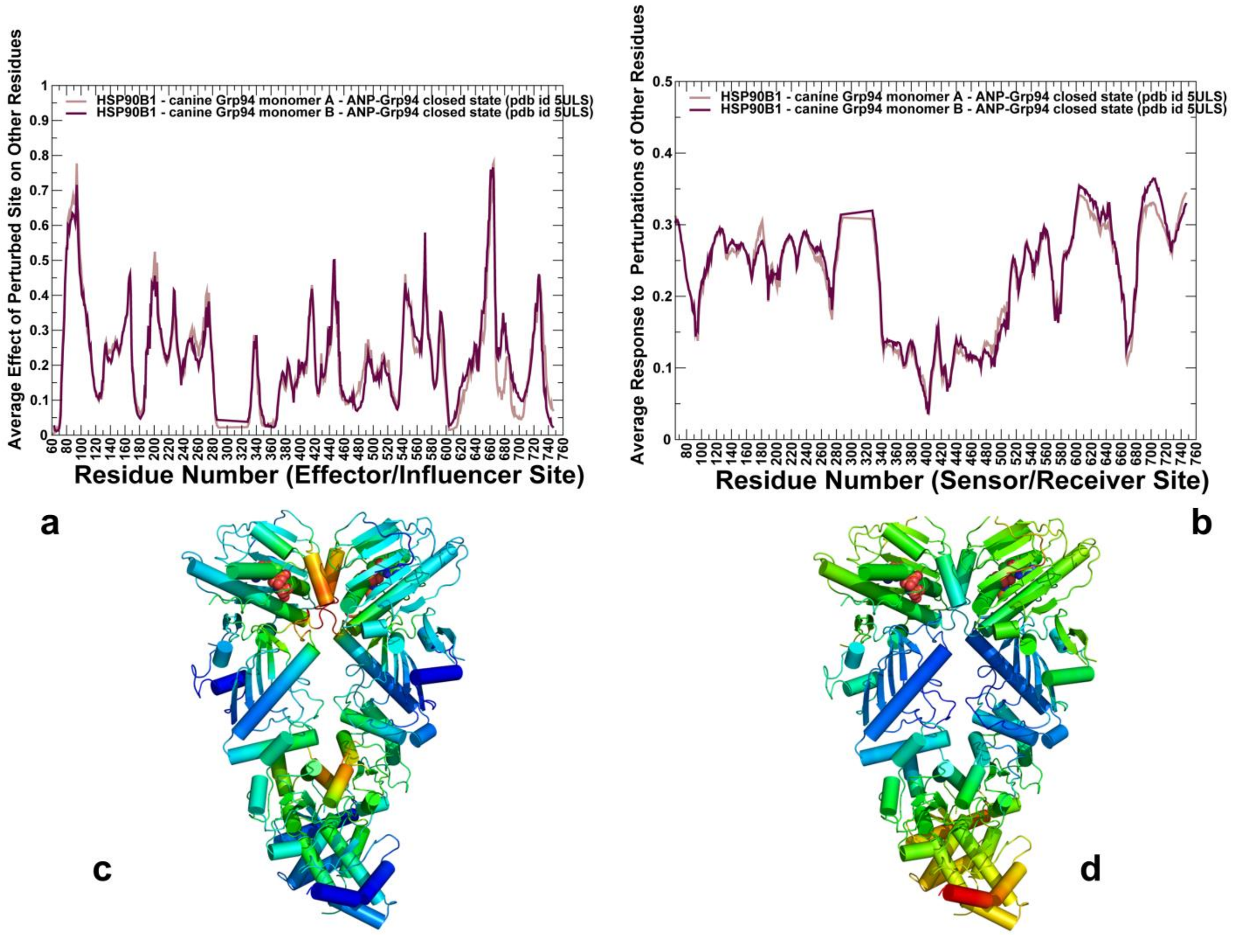

Supplementary Figure S8. The PRS Analysis of the Closed Grp94 Dimer. (a) The residue-based effector profile measures the average impact of the perturbed site on all other residues in the closed Grp94 dimer structure. (b) The residue-based sensor profile measures the average response of a given residue to perturbations of all other residues in the closed Grp94 dimer. In panels (a,b) the effector/sensor profiles are shown for monomer A in light brown lines and for monomer B in maroon lines. (c) The crystal structure of the closed Grp94 dimer form (pdb id 5ULS) is color-coded by ability to propagate perturbations, where red regions are strongest effectors. (d) The crystal structure is color-coded by sensitivity to perturbation. Red/orange regions are the most susceptible sensor sites, while dark-blue regions are the most insensitive sites; green/cyan regions show moderate sensitivity to perturbations.

Supplementary Table S1. The List of Studied PTM Sites in the yeast Hsp82 (UNIPROT HSP82_YEAST)

**Position**	**Amino Acid**	**Modification**	**Spectral Count**
22	T	phosphorylation	7
24	Y	phosphorylation	7
27	K	acetylation	7
54	K	ubiquitination	1
258	K	acetylation	7
259	K	acetylation	7
282	S	phosphorylation	1
297	S	phosphorylation	2
379	S	phosphorylation	1
484	K	acetylation	7
533	T	phosphorylation	1
547	Y	phosphorylation	1
551	T	phosphorylation	1
601	S	phosphorylation	1
602	S	phosphorylation	2
604	S	phosphorylation	1
612	T	phosphorylation	1
619	S	phosphorylation	1

Supplementary Table S2. The List of Studied PTM Sites in the human Hsp90β (UNIPROT HS90B_HUMAN)

Position	Amino Acid	Modification	Spectral Count
31	T	phosphorylation	7
33	Y	phosphorylation	7
36	K	acetylation	7
45	S	phosphorylation	1
48	S	phosphorylation	1
53	K	ubiquitination	1
56	Y	phosphorylation	9
64	K	ubiquitination	6
64	K	acetylation	1
67	S	phosphorylation	1
69	K	ubiquitination	6
95	K	ubiquitination	3
95	K	acetylation	7
107	K	ubiquitination	3
148	K	ubiquitination	3
180	K	ubiquitination	1
186	K	ubiquitination	6
186	K	acetylation	5
192	Y	phosphorylation	27
199	K	acetylation	8
203	K	acetylation	8
204	K	ubiquitination	2
204	K	acetylation	10
219	K	ubiquitination	2
219	K	acetylation	4
226	S	phosphorylation	21
237	K	ubiquitination	2
255	S	phosphorylation	33
261	S	phosphorylation	12
263	K	acetylation	6
265	K	acetylation	6
275	K	ubiquitination	2
275	K	acetylation	2
276	Y	phosphorylation	10
284	K	ubiquitination	8
284	K	acetylation	1
285	T	phosphorylation	1
286	K	ubiquitination	8
286	K	acetylation	7
297	T	phosphorylation	1

301	Y	phosphorylation	1
306	K	ubiquitination	7
306	K	acetylation	1
307	S	phosphorylation	2
309	T	phosphorylation	1
319	K	ubiquitination	6
347	K	ubiquitination	3
347	K	acetylation	7
354	K	ubiquitination	6
354	K	acetylation	1
391	S	phosphorylation	1
399	K	ubiquitination	10
399	K	acetylation	1
402	K	acetylation	2
406	K	acetylation	7
410	K	acetylation	7
411	K	ubiquitination	2
423	K	acetylation	8
427	K	acetylation	8
428	K	ubiquitination	6
428	K	acetylation	8
435	K	ubiquitination	9
435	K	acetylation	2
438	K	ubiquitination	6
445	S	phosphorylation	1
452	S	phosphorylation	3
477	K	ubiquitination	2
477	K	acetylation	4
481	K	ubiquitination	6
481	K	acetylation	11
482	S	phosphorylation	1
484	Y	phosphorylation	35
485	Y	phosphorylation	14
491	K	ubiquitination	2
491	K	acetylation	10
526	K	ubiquitination	3
531	K	ubiquitination	4
532	S	phosphorylation	3
538	K	ubiquitination	4
538	K	acetylation	8
550	K	ubiquitination	1
550	K	acetylation	1
559	K	ubiquitination	3
565	K	ubiquitination	2
568	K	ubiquitination	1

568	K	acetylation	2
574	K	acetylation	7
577	K	ubiquitination	2
577	K	acetylation	7
596	Y	phosphorylation	28
607	K	ubiquitination	7
619	Y	phosphorylation	27
623	K	ubiquitination	6
623	K	acetylation	1
624	K	ubiquitination	2
624	K	acetylation	6
641	K	acetylation	7
649	K	ubiquitination	3
649	K	acetylation	6
685	K	ubiquitination	2
718	S	phosphorylation	1

Supplementary Table S3. The List of Studied PTM Sites in the canine Grp94 (UNIPROT ENPL_CANLF)

Position	Amino Acid	Modification	Spectral Count
75	K	ubiquitination	3
75	K	acetylation	1
92	S	phosphorylation	1
94	Y	phosphorylation	1
95	K	ubiquitination	2
97	K	ubiquitination	2
97	K	acetylation	7
106	S	phosphorylation	1
109	S	phosphorylation	1
142	K	ubiquitination	1
161	K	ubiquitination	1
165	T	phosphorylation	1
168	K	ubiquitination	2
179	T	phosphorylation	1
200	Y	phosphorylation	1
201	S	phosphorylation	1
252	K	ubiquitination	1
265	K	ubiquitination	1
270	K	ubiquitination	2
270	K	acetylation	7
306	S	phosphorylation	4
347	S	phosphorylation	1
360	K	ubiquitination	2
392	S	phosphorylation	1
405	K	ubiquitination	2
410	K	ubiquitination	1
428	K	acetylation	5
434	K	ubiquitination	5
434	K	acetylation	5
455	K	ubiquitination	1
467	K	ubiquitination	1
468	T	phosphorylation	1
473	K	ubiquitination	2
486	K	ubiquitination	1
586	K	ubiquitination	1
593	K	ubiquitination	2
613	K	acetylation	1
633	K	ubiquitination	1
652	Y	phosphorylation	1
663	K	ubiquitination	3

671	K	ubiquitination	4
677	Y	phosphorylation	2
678	Y	phosphorylation	1
682	K	ubiquitination	1
682	K	acetylation	1
684	T	phosphorylation	1
733	K	ubiquitination	1
786	T	phosphorylation	2
